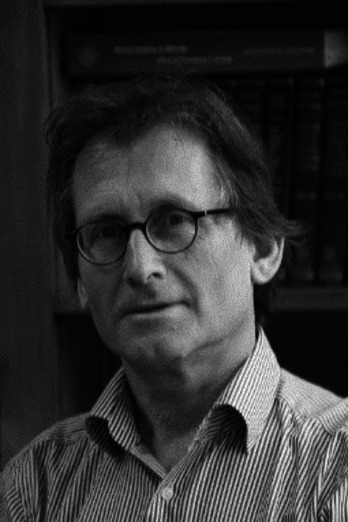# Chemically Driven Rotatory Molecular Machines

**DOI:** 10.1002/anie.202206631

**Published:** 2022-09-05

**Authors:** Anirban Mondal, Ryojun Toyoda, Romain Costil, Ben L. Feringa

**Affiliations:** ^1^ Stratingh Institute for Chemistry University of Groningen Nijenborgh 4 9747 AG Groningen The Netherlands; ^2^ Department of Chemistry Graduate School of Science Tohoku University 6-3 Aramaki-Aza-Aoba Aobaku, Sendai 980-8578 Japan

**Keywords:** Chemically Driven Molecular Machines, Molecular Motors, Molecular Rotors, Molecular Switches

## Abstract

Molecular machines are at the frontier of biology and chemistry. The ability to control molecular motion and emulating the movement of biological systems are major steps towards the development of responsive and adaptive materials. Amazing progress has been seen for the design of molecular machines including light‐induced unidirectional rotation of overcrowded alkenes. However, the feasibility of inducing unidirectional rotation about a single bond as a result of chemical conversion has been a challenging task. In this Review, an overview of approaches towards the design, synthesis, and dynamic properties of different classes of atropisomers which can undergo controlled switching or rotation under the influence of a chemical stimulus is presented. They are categorized as molecular switches, rotors, motors, and autonomous motors according to their type of response. Furthermore, we provide a future perspective and challenges focusing on building sophisticated molecular machines.

## Graphical abstract

The possibility of generating unidirectional, controlled rotary motion by chemical conversion is a major challenge. This Review provides an overview of approaches to the design, synthesis, and dynamic properties of various classes of atropisomers that can perform controlled switching or directional rotational motion under the influence of a chemical stimulus. Future strategies and challenges for the development of autonomous molecular motors are also provided.

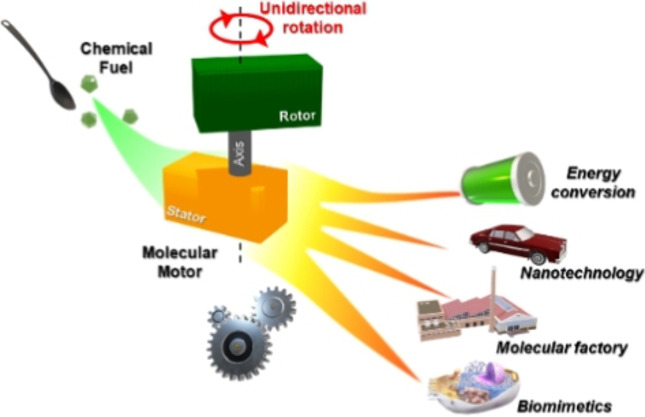

## 1 Introduction

In his lecture *“There's Plenty of Room at the Bottom*”, Richard Feynman posed the challenge of building a working electric motor that would fit into a 1/64‐inch cube.^[1]^ Although he did not have to wait long, his desire to think small laid the foundation for the modern era of nanotechnology and set the stage for scientists to control motion at the molecular level. Since then, numerous artificial molecular machines have been designed and synthesized,^[2]^ one of which is also powered by electricity.^[3]^ One of the noteworthy discoveries is the unidirectional light‐driven rotatory motor based on an overcrowded alkene developed by our group.^[4]^ The combination of photo‐induced isomerization and thermal helix inversion processes results in unidirectional rotary cycles. Following this discovery, various structural modifications have resulted in the molecular engineering of generations of light‐driven molecular motors,^[5]^ and their unique motions have been exploited in many potential future applications such as transporters, i.e., a molecular nanocar,^[6]^ supramolecular assemblies,^[7]^ responsive materials,^[8]^ functional surfaces,^[9]^ chiral catalysts,^[10]^ and bio hybrid systems.^[11]^ Apart from the artificial molecular machines, Nature produces its own machines that are responsible for muscle contraction, motility, cellular cargo transportation, and ATP fuel production among others. Myosins, kinesins, the bacterial flagellar motor and dyneins are well‐known examples where motor proteins operate efficiently to sustain fundamental processes of life.^[12]^ It is noteworthy to mention that these biological motors consume chemical energy derived from the hydrolysis of adenosine triphosphate (ATP) for their mechanical work.^[13]^ Another category of biological rotatory motors is ATP synthase, a membrane‐spanning protein which catalyzes ATP formation from adenosine diphosphate (ADP).^[14]^ Its rotatory motion, which is an essential process to produce ATP, is powered by the flow of hydrogen ions across the membrane.^[15]^ As we can learn from these sophisticated biological molecular machines, conversion of chemical fuels is an effective strategy for inducing continuous molecular motions and mechanical function in living systems. Taking this as an inspiration, various research programs have been initiated for the development of artificial molecular machines driven by chemical stimuli.[^2^, ^5a^, ^16^] We believe these biomimetic systems have a lot of potential for realizingfuture molecular machinery. In this Review, we highlight the most recent developments of chemically driven molecular machines that perform directional rotation around a single covalent bond.

**Figure 1:**
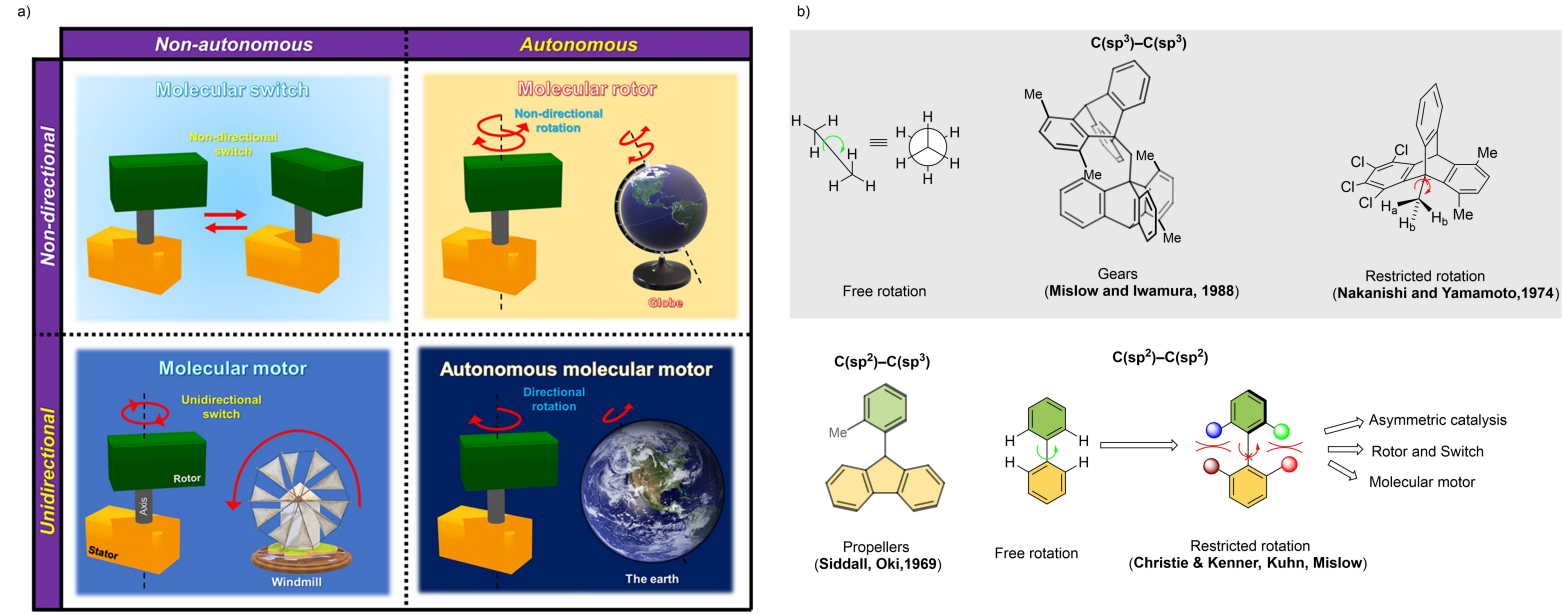
a) Schematic illustrations of different controlled movements and types of molecular machines. They consist of a rotator (green) and a stator (orange) which are held together with an axle (black). b) Illustrative examples of the early pioneering developments of restricted molecular rotation about a C−C single bond which resulted in various applications in the area of asymmetric catalysis and molecular machines.

Before going into detail, we define here the terminology used in this Review (Figure 1). In recent years, there has been an increasing interest in developing synthetic molecular machines that mimic aspects of biological machines. These machines can serve as platforms for the creation of artificial molecular systems including molecular assemblers, walkers, and pumps. In this Review, we focus on a fascinating class of artificial molecular machines based on atropisomers, which convert chemical energy into rotational motion around single bonds. They are categorized into molecular switches, rotors, and motors, and comprise a rotating unit (rotator) and a stationary part (stator) which are interconnected by an axle (Figure 1a). In a molecular switch, the rotator unit can reversibly change its position between two or more stable states (Figure 1a, top left). This switching can happen when an external stimulus (such as light, chemical fuel, temperature, or electricity) is applied. In contrast, a molecular rotor performs continuous free rotation at room temperature (Figure 1a, top right).

In this case, the rotation is considered to be “free” and nondirectional, and, in some cases the rotational speed can also be regulated by external chemical stimuli. When control of directionality is added to the motion of molecular switches and 360° clockwise or counter‐clockwise rotation can be achieved, we call such unidirectional switches molecular motors (Figure 1a, bottom left). Unidirectional rotation becomes possible, for instance, when a chiral auxiliary is present in the close vicinity of the motor axle by creating a diastereomeric relationship between forward and backward rotation (see below).^[17]^ Similarly, we can define a unidirectional rotor as an autonomous molecular motor (like the rotating Earth) (Figure 1a, bottom right). An autonomous molecular motor functions by inducing a net direction of rotation as long as fuel (chemicals) is present. Therefore, sequential addition of chemicals is not necessary. As mentioned earlier, many protein‐based biological molecular motors undergo autonomous motion using the chemical energy released by the hydrolysis of ATP to perform mechanical work. Therefore, we believe that these autonomous, synthetic, chemically driven molecular motors will find widespread applications as machines in molecular nanotechnology. Achieving comparable autonomous operation in synthetic systems therefore remains a goal of paramount importance. During autonomous operation, the ratchet mechanism must operate continuously to ensure smooth operation while a chemical fuel is consumed. To avoid violation of the second law of thermodynamics, the barriers must therefore be raised and lowered repeatedly under the same reaction conditions.^[5a]^ Finally, we define a chemical fuel as a reagent that reacts with a molecular machine in the process of switching or rotation, i.e., chemically driven conformational change.^[18]^

In order to control the rotational barrier, rate, and direction of rotation about a single covalent bond, atropisomers are often selected as the basis, owing to their restricted rotation around the axle. The term “atropisomerism” was first introduced by Kuhn in 1933,^[19]^ but the existence of axial chirality was already discovered in 1922 by Christie and Kenner.^[20]^ Pioneering work on optical activity as a consequence of restricted rotation of biphenyls was done by Mislow and co‐workers.^[21]^ In contrast to ethane, which undergoes free rotation around its C(sp^3^)−C(sp^3^) bond at room temperature due to the low rotational barrier, restricted rotation can be observed around a crowded C(sp^3^)−C(sp^3^) as well as C(sp^2^)−C(sp^3^) bond (see Figure 1b). Following the pioneering work by Siddall, Oki, Mislow, Iwamura, Nakanishi, Yamamoto,^[22]^ and others,^[23]^ a number of molecular gears^[24]^ and propellers^[25]^ were developed. The importance of axially chiral compounds has drastically changed since 1980, when BINAP was introduced by Noyori for the asymmetric hydrogenation of olefins.^[26]^ Following this breakthrough discovery, numerous ligands and catalysts with axially chiral biaryl backbones have been described for asymmetric transformations.^[27]^ The configurational stability and the rotation about the chiral axle of atropisomers depend on several factors such as 1) the bulkiness parameter (defined by A‐value) of the substituents closest to their rotational axle;^[28]^ 2) the existence of a bridge which interconnects the *ortho*‐substituents;^[29]^ 3) a photochemical or chemically induced process that facilitates atropisomerization.^[30]^ Taking into account these key factors, this Review aims to highlight various designs, structural features, and operational mechanisms of distinct artificial molecular machines where chemical responses (such as hydrogen bonding, metal coordination, pH changes, and covalent bond formation) cause rotatory movements. Our discussion starts with nondirectional chemically driven molecular switches and rotors. Later we give an overview of the design and synthesis of unidirectional molecular motors, including a recent study reporting the first autonomous molecular motor. Finally, a future perspective for the development of autonomous molecular motors is presented. We believe biaryl‐type chemically driven molecular motors are a unique class of molecular machines and due to their rotatory mechanism, involving a single biaryl axle, highly distinctive from other motor designs. The control of directional rotation around the biaryl using chemical transformations shows not only the simplicity and elegance in designing motors by controlling rotation around a single C−C bond but also the fundamental differences in approach compared to simple rotor functions and other mechanical machines. Moreover, tuning the rotation rate of an aryl ring, i.e., atropisomerization, is possible simply by chemical modification of the *ortho*‐substituents. There is an abundance of work dedicated to the synthesis of biaryl structures that makes their design and synthesis more straightforward. This field of research is at the earliest stage and opens up the prospect of future synthetic machineries. It is worth noting that there are sophisticated designs for chemically driven molecular machines other than axially chiral molecular systems, for example, mechanically interlocked catenanes,^[31]^ ferrocene‐containing molecular switches and rotors,^[32]^ and gear‐shaped metal porphyrin based rotors^[33]^ which are discussed in detail in several excellent reviews.^[34]^ In addition to the chemically induced rotation, control of movements around C−C single bonds of biaryls has also been realized using an external photoswitchable group attached to the molecule.^[35]^

Here we also describe the methods and experimental techniques that can be used to study mechanisms of molecular machines. While an increasing number of analytical methods have been developed and can be applied to investigate axially chiral motors and switches, we focus on several primary ones as this section is primarily intended for those starting in this field of research. Nuclear magnetic resonance (NMR) spectroscopy is the most widely used analytical technique for understanding the switching process, provided that the individual states of the switching process are diastereomeric. Diastereoisomers exhibit diagnostic differences in their NMR spectra so that the extent of the chemical switching can be readily determined. NMR techniques using ^1^H, ^13^C, ^19^F, and ^31^P nuclei are often used for this purpose.

HPLC (High‐performance liquid chromatography) with chiral stationary phases is known to be effective for the separation of enantiomers as well as diastereoisomers. HPLC can also be used to determine switching ratios of molecular machines, since different conformations of a motor or a switch can exhibit different retention times. If a chemical waste is generated during the switching process, it may interfere with the NMR spectrum but HPLC analysis may be utilized even in this case for tracing molecular switching. Chiral HPLC is also an excellent tool for analyzing isomers (like atropisomers) when a compound is chiral and cannot be analyzed by NMR spectroscopy.

Single‐crystal X‐ray crystallography is the most powerful of all techniques as it allows the determination of three‐dimensional (3D) structures of molecules in their solid state. Despite the advantages of this method, its main drawback is that it requires a single crystal of a compound, which is typically difficult to obtain, in particular for compounds containing thermally labile chiral information. Nevertheless, if a suitable single crystal for a molecular machine is obtained for each conformational switching state, its rotation process can be visualized clearly.

Another technique is electronic circular dichroism (CD) spectroscopy. CD spectroscopy is often used to determine the helicity of atropisomeric biaryls.

## 2 Chemically Driven Molecular Switches

In the following section, various types of molecular switches will be discussed. They are categorized according to the following principles based on the parameters used to achieve conformational changes, i.e., molecular switching: 1) through modulation of hydrogen‐bonding; 2) solvent‐assisted conformational control; 3) pH‐responsive switching in biphenyl; and 4) through ion binding.

### 2.1 Conformational Switching Controlled by Intra/Intermolecular Interactions

Hydrogen bonds provide a unique electrostatic noncovalent interaction between a hydrogen atom (that is covalently bound to a more electronegative atom) and an electronegative atom bearing a pair of electrons (such as nitrogen, oxygen, and halogens).^[36]^ They are categorized as weak bonds with a bond strength that is adequate for modulating molecular geometries and assemblies.^[37]^ Through the process of evolution, Nature has created various nanoscale self‐assembled H‐bond‐based systems to achieve functions essential for life. Prominent among these are the proteins as well as the double‐stranded DNA helices which are controlled by the presence of multiple hydrogen bonds between Watson–Crick base pairs.^[38]^ Taking inspiration from biological systems, various sophisticated artificial molecular systems have been constructed where hydrogen bonds are used for creating supramolecular assemblies,^[39]^ engineering molecular packing in crystals,^[40]^ and tuning material properties.^[41]^ Over the past decades, hydrogen bonds have played an important role in the design of artificial molecular systems and several molecular machines that exhibit rotation upon manipulation of this weak interaction were developed.^[42]^ Here, we first introduce various well‐designed examples of molecular switches, where the precise control of hydrogen bonding is used for their conformational changes.

**Scheme 1:**
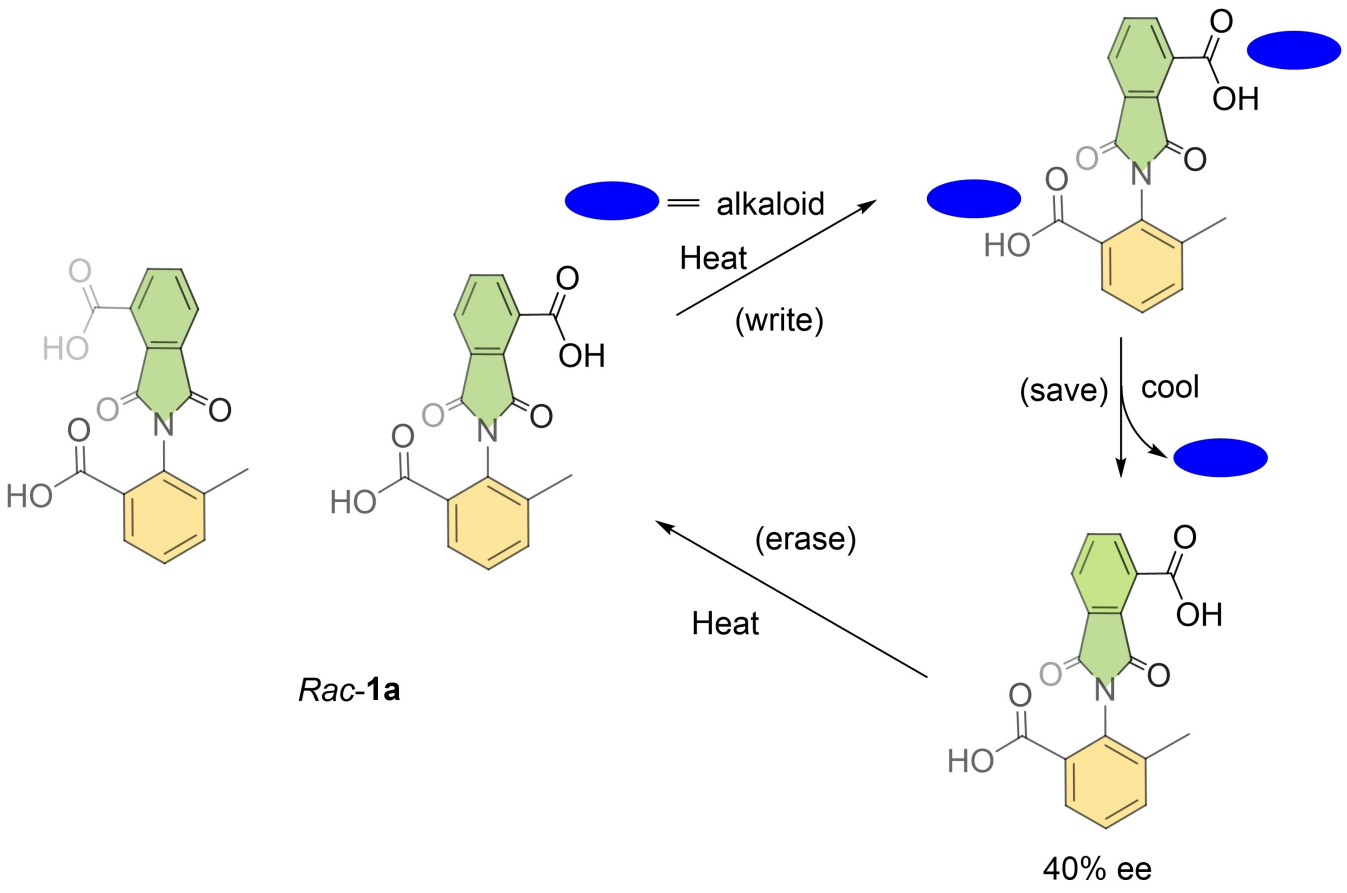
Molecular switch ***Rac*** **‐1 a** regulated by intermolecular H‐bonds.^[43]^

In 2009, the group of Shimizu reported an axially chiral diacid switch ***Rac*** **‐1 a** as shown in Scheme 1.^[43]^ Rotation around the central C−N single bond is hindered due to the intrinsic high rotation barrier at room temperature. When the diacid is heated at 100 °C, the rotation around the single bond becomes feasible since the associated activation energy for the rotation is overcome. Upon addition of a chiral Lewis basic guest such as quinine and quinidine, the diacid forms a diastereomeric complex through hydrogen bonding between the amine and the carboxylic acid. A directional bias can be created by transferring the chirality of the guest to the diacid, producing one of the stereoisomers preferably. Then, when the molecular switch is cooled to room temperature, the C−N bond rotation is restricted again while keeping the chiral information intact. After removal of the chiral guest, the original racemic state can be reobtained by heating the diacid at high temperature. This reversible chiral switch is robust and writing/erasing is repeatable as these processes are carried out using noncovalent interactions and thus the molecular complex can be applied as a chiral memory device.

**Scheme 2:**
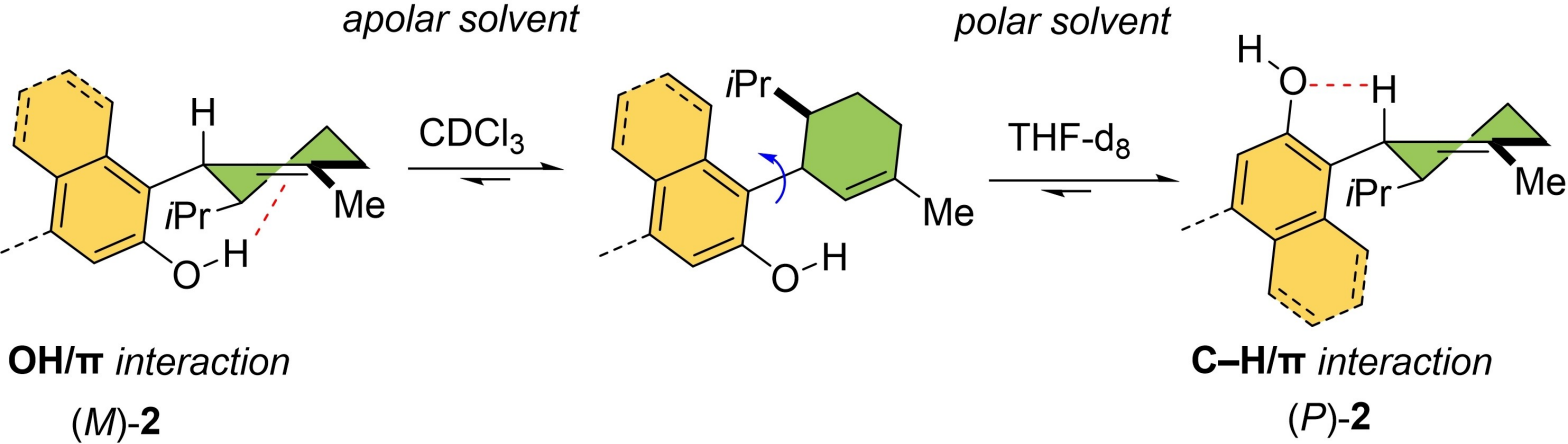
Conformational control of a cannabidiol derivative by solvent polarity.^[44]^

A combination of noncovalent interactions such as hydrogen bonding and OH/π interaction has been used to control the axial conformation of cannabidiol derivative **2**, by simply tuning the solvent polarity (Scheme 2).^[44]^ NMR studies together with theoretical calculations revealed that an intramolecular OH/π bond is the key interaction to induce the *M* conformation in an apolar solvent (chloroform). On the other hand, in a polar solvent (THF), CH/O hydrogen bonds are proposed to become dominant to afford mainly the *P* conformation.

**Scheme 3:**
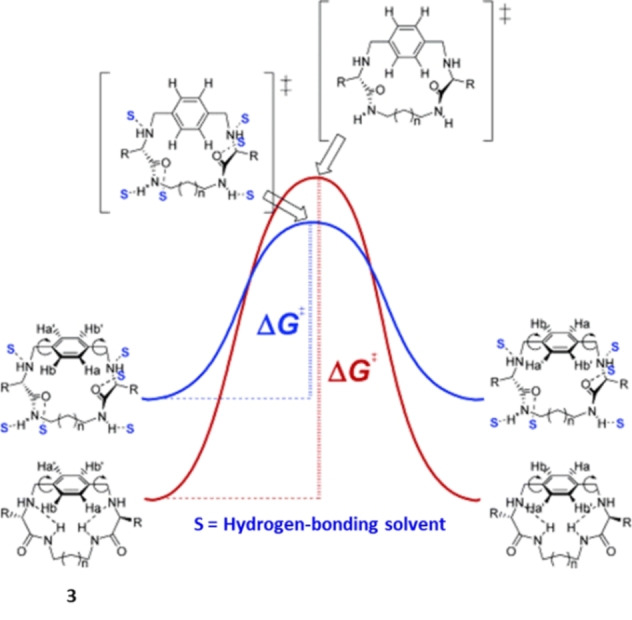
The effect of polar solvents on the energy barrier of the aromatic ring rotation of peptidomimetic cyclophanes. Reproduced with permission from ref. [45] Copyright 2006, American Chemical Society.

Hydrogen bonding is sometimes strongly dependent on the solvent–solute interaction.^[45]^ The group of Luis synthesized peptidomimetic cyclophanes and, through variable‐temperature NMR studies and theoretical modelling, noticed that the aromatic ring shows distinct rotary behavior in different solvents (Scheme 3). In an apolar solvent, the rotor shown in Scheme 3 has a preference for a conformation in which intramolecular hydrogen bonds stabilize the ground state, and has a high energy barrier for the rotation of the aromatic ring. In contrast, a polar solvent can break the intramolecular hydrogen bonds and solvate both the ground state and the transition state effectively, reducing the rotational barrier (Scheme 3). Taking advantage of this system, the authors demonstrated control of the rotation rate by introducing methanol as an additive to a chloroform solution of the compound. Due to the increased solvent polarity, threefold accelerated rotation was achieved in the presence of 5 % v/v of methanol.

In addition, it is worth noting that the Aprahamian group developed hydrazone‐based acid/base‐activated switches that show *E*/*Z* isomerization by controlling the hydrogen bonding.[^46^, ^47^]

### 2.2 pH‐Induced Molecular Switches

pH‐Induced molecular conformational changes have been an extensive research topic since the phenomena are similar to what is observed in biological systems.^[48]^ In this section, several advances in controlling molecular rotation around a C−C bond by addition of acid or base are presented.

**Scheme 4:**
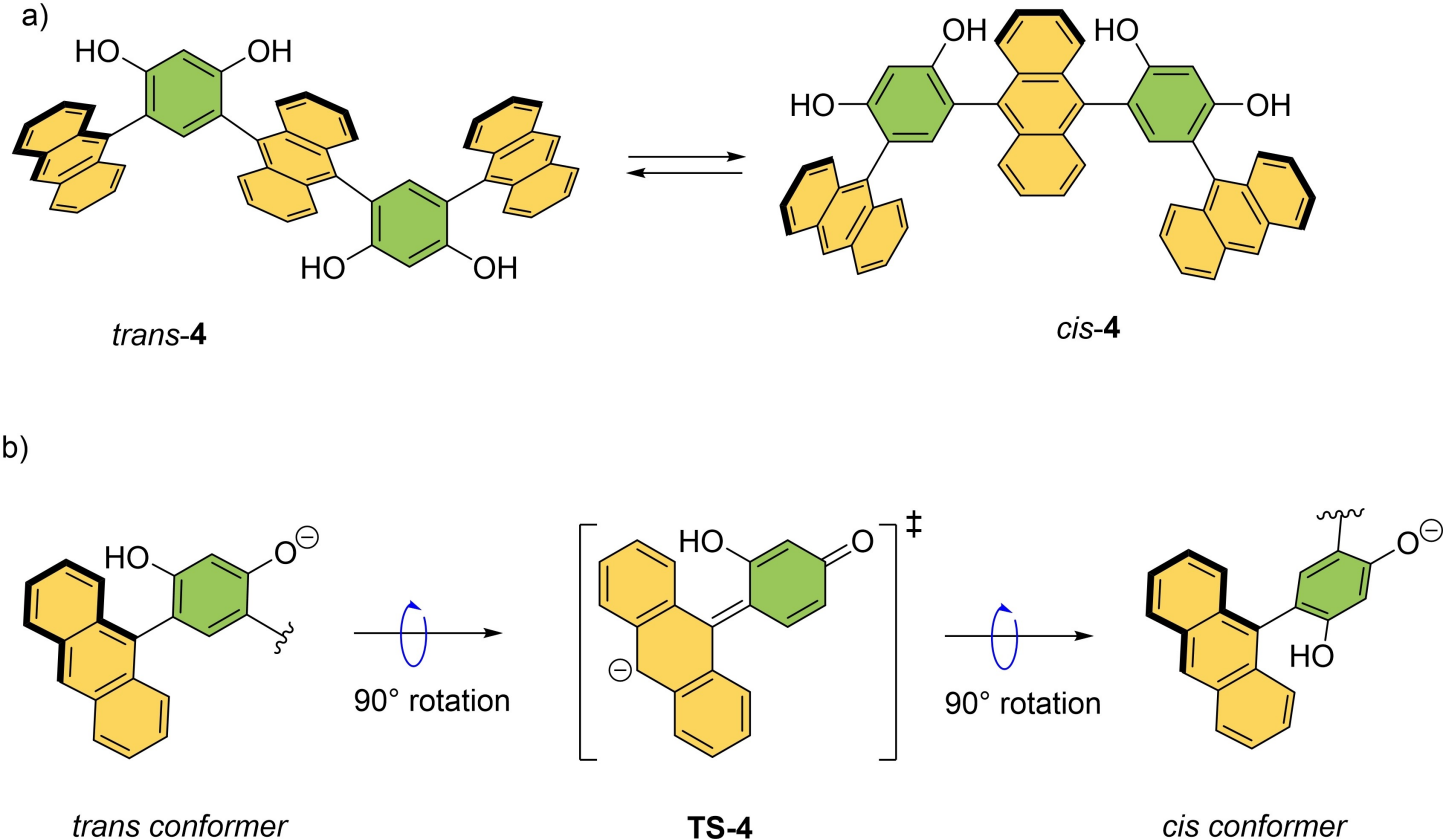
a) Base‐controlled switching of molecular conformation by controlling rotation in anthracene trimer **4**. b) Mechanism of the *cis*–*trans* isomerization via a quinoid‐type planar conformation.^[49]^

An excellent example of a pH‐responsive switch was developed by Yoshizawa and co‐workers.^[49]^ A selective *cis*–*trans* isomerization through rotation around an aryl–aryl bond was achieved in an anthracene trimer‐based switch upon addition of a base (*trans* **‐4**, Scheme 4a). Compound **4**, featuring four biaryl single bonds, was obtained in its pure atropisomeric form in four synthetic steps. At room temperature, atropisomerization is restricted due to the steric hindrance imposed by the *ortho*‐substituents in the central aromatic units. However, upon deprotonation of one of the hydroxyl groups with a base (NaOH), *trans*‐anthracene trimer **4** can be selectively transformed to its *cis* isomer (with the *cis/trans* ratio of 9 : 1) through 180° rotation around the single bond. Mechanistically, this rotation around an aryl–aryl bond occurs via a planar transition state as shown in Scheme 4b. A planar quinoid‐type transition state, i.e., **TS‐4** is stabilized through extended conjugation between the two aromatic rings when one of the hydroxyl groups gets deprotonated upon addition of a base.

As a result, the barrier of rotation is greatly reduced and favors the formation of the *cis*‐isomer. Similar *cis–trans* isomerization is also observed in basic methanol solution, albeit with rather poor selectivity (*cis* : *trans* 2 : 3). According to the authors, the change in isomer ratio can be attributed to the hydrophilic nature of the exterior of the molecule due to the alignment of multiple hydroxyl groups in the *cis*‐form, which is stabilized by solvent–solute interaction when water is used as a solvent, favoring the formation of the *cis*‐isomer.

**Scheme 5:**
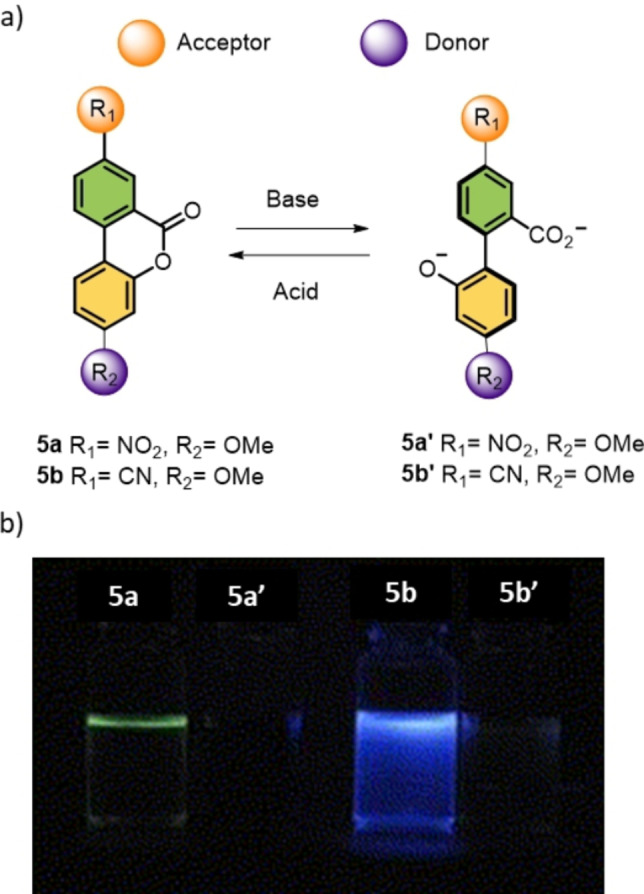
a) Schematic representation of a pH‐driven biaryl switch. b) Change in photoluminescence upon switching. Reproduced with permission from ref. [50] Copyright 2012, Elsevier.

A different design of a biaryl‐based switch which shows variable photoluminescence catalyzed by both acid and base was developed by Dahl and co‐workers (Scheme 5).^[50]^ The key feature of this design is that the luminescence of the biaryl lactone **5 a/5 b** can be switched ON and OFF by a reversible conversion between the open and the closed form as shown in Scheme 5a, with the help of an external acid or a base.^[50]^

Luminescence derived from intramolecular charge transfer (ICT)^[51]^ in organic molecules is of particular importance in the development of advanced optical materials.^[52]^ The degree of charge transfer in a π‐conjugated donor (D)–acceptor (A) dyad (D−π−A) is heavily influenced by the extent of π‐conjugation, and acid/base‐responsive structural change may alter the photoluminescence properties. Biaryl switch **5** with a D−π−A interaction between R_1_ and R_2_ in the planar state results in an intense ICT band (356 nm for **5 a** and 317 nm for **5 b**) due to the π orbital overlap which facilitates the electron transport between the two aryl rings. However, upon hydrolysis with a base (tetrabutylammonium hydroxide), diminution of the ICT band is observed due to formation of a nonplanar dianionic carboxylate (**5 a′** and **5 b′**, Scheme 5a). This is attributed to the partial reduction of conjugation between the aryl rings where the aryl rings are lying out of the plane (partially twisted) in order to mediate an anion–anion repulsion and steric hindrance. Upon addition of an acid, a ring‐closing reaction reformed the planar form. This switching behavior was also monitored with the associated photoluminescence properties. As shown in Scheme 5b, lactones **5 a** and **5 b** in the planar form displayed faint green and light blue luminescence, respectively, which can be quenched by addition of a base. As expected, the planar lactones reformed upon addition of an acid (HCl or TFA), recovering the original photoluminescence.

**Scheme 6:**
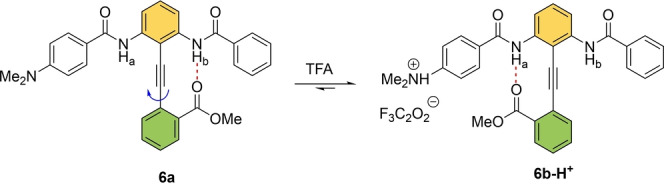
A 180° rotation about the acetylene axis by modulation of the H‐bonding interaction.^[53]^

Hamilton and co‐workers introduced a pH‐responsive biaryl‐type conformational switch based on a multifunctional diphenyl‐acetylene **6** (Scheme 6).^[53]^ In solution (CD_2_Cl_2_, 298 K), the orientation of the ester group at the lower half gives two possible conformations for **6**. Compound **6 a** has the ester group H‐bonding to H_b_ and **6 b** to H_a_. The electron‐donating character of *p*‐NMe_2_ weakens the H‐donating capabilities of H_a_, shifting the equilibrium towards **6 a** with a **6 a** : **6 b** ratio of 1.3 : 1. This is due to the strong H‐bonding between the C=O of the ester group and H_b_. In contrast, the electron‐donating ability of *p*‐NMe_2_ can be quenched via protonation upon addition of trifluoroacetic acid (TFA), which resulted in almost complete conversion towards **6 b‐H^+^**, i.e. the protonated form of **6 b**. The authors further extended their design towards a series of benzamido‐diphenylacetylene (DPA) molecular switches where the relative hydrogen‐bonding capabilities of two amide groups determine the extent of conformation switching.^[54]^

**Scheme 7:**
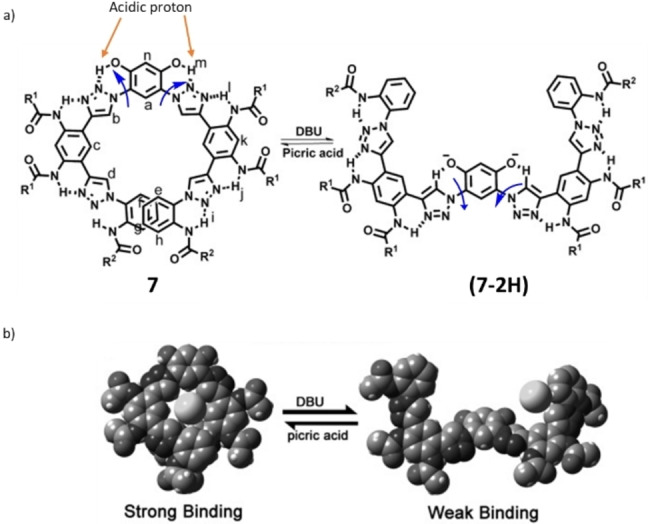
a) An acid/base‐mediated uptake and release of halide ion through a conformational switching. b) An illustration of halide anions being taken up and released when switching between **7** and (**7‐2H**). Reproduced with permission from ref. [58] Copyright 2015, Wiley‐VCH.

Allosteric regulation of binding sites in biological machines is of high importance because more dynamic and complex control of the enzyme activity is permitted (Scheme 7).^[55]^ Because of the binding of cations and anions to the receptors, these machines can also be used in sensing, self‐assembly, extraction, transport, and catalysis. In order to mimic the biological ion pumps, and following pioneering studies of Shinkai on azobenzene‐based receptors,^[56]^ several photoswitches were developed with the ability to uptake and release various cations and anions.^[57]^ As an alternative approach, Zhao and co‐workers reported the design of an aryl‐triazole foldamer **7**, in which an acid/base‐mediated uptake and release of halide (mainly chloride) anions occur with high selectivity, through a rotation around the C−N single bonds.^[58]^ As shown in Scheme 7b, helical foldamer **7**, owing to its close‐shaped cavity, displays a strong affinity towards halide anions. Upon deprotonation of the resorcinolic O−H group with an organic base (1,8‐diazabicyclo[5.4.0]undec‐7‐ene, DBU), foldamer **7** unfolds into an open conformation (**7‐2H**) through the modulation of its intramolecular hydrogen‐bonding network. In the open conformation (**7‐2H**), due to the electrostatic repulsion between the halide anion and the resorcinolate oxyanions, the halide is released (Scheme 7b). This simple acid/base‐triggered conformational switch provides a basis for the future design of chemical‐stimuli‐driven receptors anion regulation.^[59]^ (For related approaches on receptor chemistry see refs. [59, 60] and for application in photoswitchable asymmetric catalysis see ref. [61]).

### 2.3 Metal‐Ion‐Coordination‐Based Molecular Switches Based on Single‐Bond Rotation

Metal ions are among the most effective additives/reagents that can facilitate controlled molecular motions (see below). In this section, illustrative examples of molecular machines showing rotatory motion around their single bonds by exploiting metal coordination are provided. Before going into details, distinctive features and advantages based on coordination complexes are noted:

1. First, coordination bonds are often dynamic and reversible in nature; therefore formation and cleavage of coordination bonds can be achieved with simple chemical manipulations such as adding a metal ion source or additional ligand, treating with base/acid, or via redox control.[^2c^, ^2e^]
2. Many coordination compounds are synthesized by hybridization of an inorganic metal ion and an organic ligand. Due to the abundant variety of these building blocks, a countless number of complexes have been studied, which enables one to select a desired motif suitable for the purpose of designing a molecular machine.
3. Metal ions often possess multiple valence states that account for their different properties. Thus, fine‐tuning of the redox state of a metal center in a complex can directly alter the stability of its coordination bonds, reactivity, stereochemistry, and also physical properties. As a result, different geometries can be achieved due to different ligand‐binding modes by shuttling between two different metal redox states.^[62]^

Due to the above‐mentioned characteristics using coordination chemistry, the installation of a metal binding site in the design of a molecular machine can be highly beneficial to manipulate nanosystems.

**Scheme 8:**
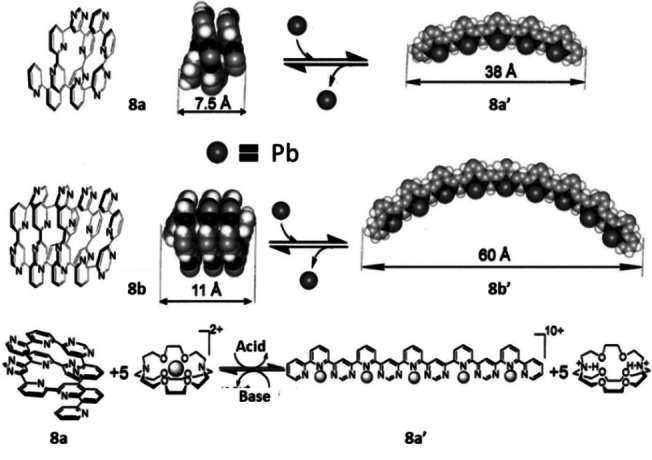
Molecular strands switchable between the extended and contracted forms. Reproduced with permission from ref. [63] Copyright 2002, National Academy of Sciences.

Lehn used metal coordination[^2c^, ^2e^, ^62^] in a supramolecular system to demonstrate chemically driven molecular extension and contraction motion via multiple C_aryl_−C_aryl_ bond rotations.^[63]^ Molecular strands featuring alternating sequences of pyridine and pyrimidine (**8 a** and **8 b**) as shown in Scheme 8 were designed. The noncoordinated N‐ligands **8 a** and **8 b** prefer the *transoid* conformations, which lead to helical structures stabilized by π–π interactions. Upon complexation with lead(II), the structure unfolds in order to adopt a tridentate binding mode, changing the conformation of the heterocyclic oligomer into a rigid and linear structure (**8 a′** and **8 b′**). In this case, the *cisoid* conformations are induced by tridentate metal ion coordination. This structural switch can be reversed by addition of a metal ion trap (cryptate), or acid and base, which extracts lead ions from the metal complex and the ligand folds in a helical structure.

**Scheme 9:**
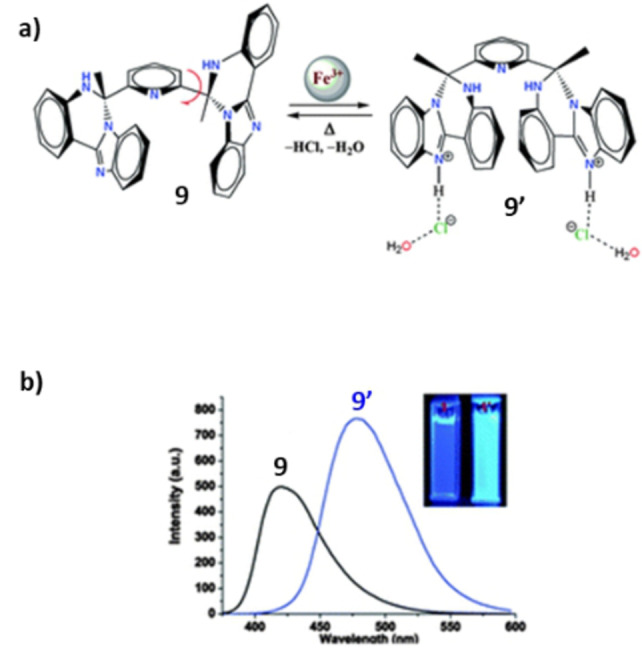
a) Fe^3+^‐sensitive molecular fluorimetric switch. b) Fluorogenic changes between **9** and **9′**. Reproduced with permission from ref. [64] Copyright 2014, Royal Society of Chemistry.

Fe^3+^‐sensitive ligand **9** comprising imidazo‐quinazoline fluorophores was introduced as a conformational switch (Scheme 9a).^[64]^ FeCl_3_ was used to induce single‐bond rotations to convert the switch to a different conformation (**9′**) which is stabilized by hydrogen‐bonding between an iminium NH and a chloride ion. The authors propose a possible mechanism of this structural switching where chloride anions generated from an aqueous solution of FeCl_3_ induce the H‐bonding interaction in **9′**.

Upon this structural change, the intense photoluminescence band associated with the complex **9′** is bathochromically shifted (Scheme 9b). As a consequence, this unique compound can function simultaneously as a conformational switch and a fluorimetric switch.

**Scheme 10:**
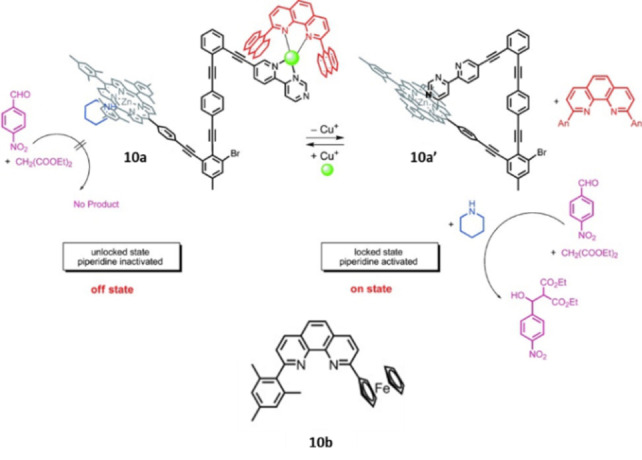
Catalytic nanoswitch regulated by metal coordination. Reproduced with permission from ref. [65] Copyright 2012, Wiley‐VCH.

Schmittel et al. developed a multifunctional and reversible bimetallic nanoswitch and applied it to switchable catalysis, mimicking the behavior of calcium/calmodulin‐dependent protein kinase II (Scheme 10).^[65]^ Molecular switch **10 a** has a zinc porphyrin core and a pyridylpyrimidine (py‐pym) unit connected to a rotatable rod. In this design, an axial zinc‐binding ligand (piperidine) and a copper‐chelating ligand (phenanthroline) are also present. With copper(I) ions, phenanthroline shields the py‐pym arm by forming a heteroleptic complex and inhibits axial coordination of pym to the zinc center. In this state, piperidine is bound to the Zn porphyrin core. When the copper ion is removed from the system by addition of a stronger ligand **10 b**, the pyrimidine nitrogen works as a stronger axial ligand for the porphyrin system and binds to the zinc center, kicking out the piperidine ligand (**10 a′**).

The authors utilized this chemical response to regulate the Knoevenagel condensation reaction in which transformation the released piperidine acts as a base catalyst (Scheme 10).

**Scheme 11:**
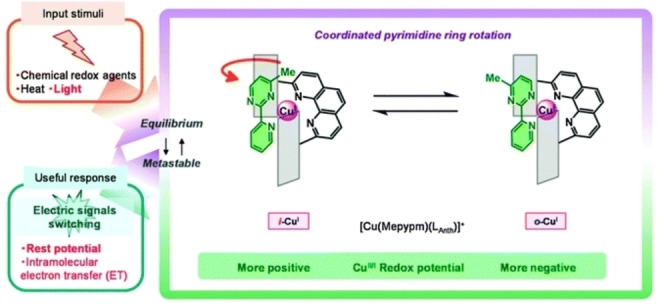
Redox‐active switch based on a copper‐bipyrimidine complex. Reproduced with permission from ref. [67d] Copyright 2013, Royal Society of Chemistry.

The redox properties of copper‐pyridylpyrimidine complexes were explored by Nishihara and co‐workers to control the rotation of a pyrimidine ring of the organic bidentate ligand.^[66]^ For example, heteroleptic copper complex **11** was prepared using a phenanthroline with bulky groups and the ring inversion of the methylpyridylpyrimidine ligand was studied. Complex **11** exists in two forms, i.e., **11 a** and **11 b**, having different biaryl geometries (Scheme 11). Through their intensive studies, the authors found that the relative stability of the *i*‐ and *o*‐isomers changes according to the oxidation state of the copper center. At the oxidized state, the Cu^II^ metal center adopts a square‐planar coordination geometry, causing steric repulsion for the *i*‐isomer. As a result, oxidation‐triggered ring rotation happens and the *o*‐isomer is preferably formed. Note that this rotation can be switched off at low temperature. Oxidation and reduction of the metal complex were achieved via a chemical approach. The complex was oxidized from Cu^I^ to the Cu^II^ state with ammonium hexanitratocerate(IV), and by addition of decamethylferrocene as a reductant the original state was retrieved with reduction of Cu^II^ to Cu^I^. Utilizing these rotamers, the electron‐transfer‐gating behavior to an electrode was successfully regulated by controlling the molecular rotation. In several follow‐up studies,^[67]^ a molecular switch with two redox centers was designed which showed regulated intramolecular electron transfer capability.^[67a]^ Another study demonstrated regulation of pyrimidine ring orientation in a heteroleptic copper complex using different solvents and counterions.^[67c]^

**Scheme 12:**
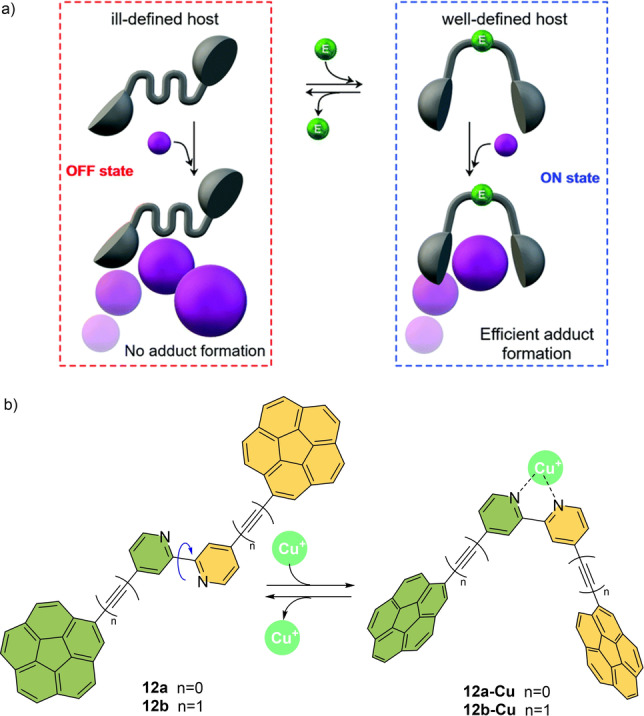
a) Blueprint of reversible molecular tweezers (black) for fullerene recognition (purple) controlled by an atropoisomerization of bpy. b) Cu^I^‐mediated reversible *syn*/*anti* conformational switching.^[68]^

Similarly, Garcia‐Rodriguez, Alvarez et al. reported a coordination/decoordination‐based switching approach to develop a molecular tweezer that can perform reversible ON/OFF recognition towards guest fullerenes.^[68]^ As depicted in Scheme 12, bpy (2,2′‐bipyridine)‐based molecular switches bearing two concave corannulene fragments whose fullerene recognition abilities can be modulated by *anti‐*to*‐syn* atropisomerization by the in situ formation of tetrahedral Cu^I^ complexes (Scheme 12). In the absence of a metal complex, the bpy scaffolds **12 a**,**b** prefer an *anti*‐conformation due to steric repulsion. In this state, the two corannulene subunits are too far apart to form a stable molecular cage with the appropriate cavity size to accommodate a fullerene unit and stabilize the inclusion complex^[69]^ (Switch‐OFF). On the other hand, the addition of [Cu(NCMe)_4_]BF_4_ and 1,2‐bis(diphenylphosphino)ethane (dppe) as secondary ligand triggered the formation of the *syn*‐conformer by a favorable C−C single‐bond rotation in the bpy unit. This is due to the stable complex formation between Cu^I^ and bidentate‐chelating bpy‐ligand. The combination of the favorably curved topology of corannulene and the *syn*‐conformation imposed by Cu^I^ leads to the generation of the stable **12 a‐Cu** or **12 b‐Cu/**fullerene complex (Switch‐ON) through a supramolecular host—guest interaction. One of the important aspects of this work is the reversibility of the switch ON/OFF process. Indeed, addition of 1 equivalent of dppe as a metal scavenger to the *syn*‐complexes **12 a‐Cu** or **12 b‐Cu** leads to the regeneration of the metal‐free *anti*‐bpy conformers **12 a** or **12 b** and releases the more stable [Cu(dppe)_2_]BF_4_ complex as a by‐product (Scheme 12b).

**Scheme 13:**
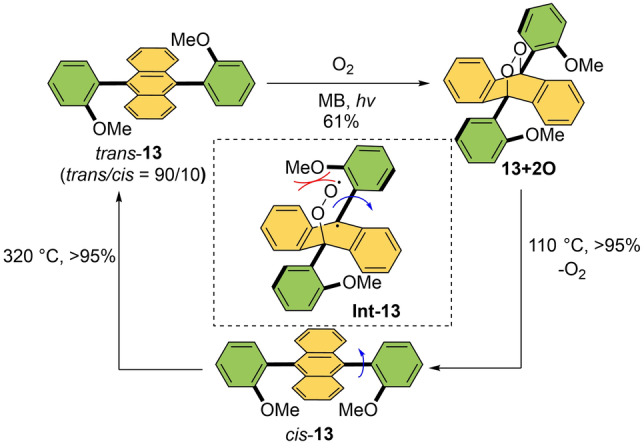
Bisarylanthracene rotary switch responsive to singlet oxygen and possible electrostatic interactions in the radical intermediate **Int‐13**.^[70]^

Singlet oxygen can be used as “fuel” in order to perform switching around the C−C bond.Linker and co‐workers developed a bisarylanthracene that works as a molecular rotary switch (**13** in Scheme13).^[70]^ Starting from more stable *trans*‐**13**, photoirradiation in the presence of oxygen and a photosensitizer generated singlet oxygen and promoted photooxygenation at the anthracene core to give endoperoxide **13+2O**. This compound exclusively exists as the *cis*‐isomer and delivered *cis* **‐13** upon deoxygenation. Based on the proposed photooxygenation mechanism,^[71]^ the authors hypothesized that electrostatic interaction between an incoming oxygen and the methoxy substituents (see Scheme 13) is the reason for the formation of the *cis*‐isomer of **13** exclusively. Heating the *cis*‐isomer produces *trans*‐**13** and consequently forces the C−C single bond to rotate, preferably forming the thermodynamically favored *trans*‐isomer with the *trans/cis* ratio of 90 : 10. Therefore, photooxygenation and thermal treatment resulted in a reversible molecular switch.

## 3 Controlling the Speed of Molecular Rotors

Manipulation of the rotational speed of molecular rotors and motors is another important goal in molecular nanoscience.[^16b^, ^72^] In this section, we will highlight single‐bond molecular rotors whose speed of rotation can be controlled by an external chemical input.

**Scheme 14:**
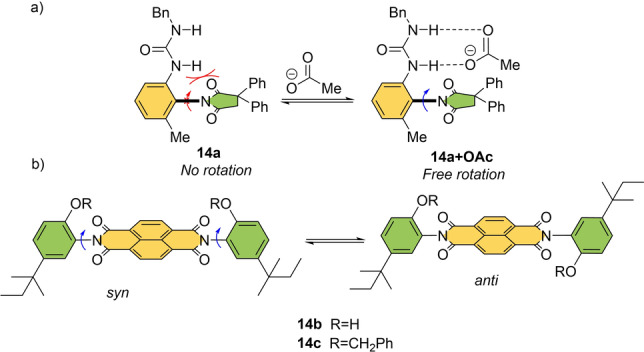
a, b) Molecular rotors regulated by hydrogen bonds.[^73^, ^74^]

Building on their studies on solvent‐controlled motion (see Scheme 1), Shimizu and co‐workers developed several molecular rotors connected by a C−N bond which have guest‐controlled tunability of their rotation rates (Scheme 14). For instance, when a urea group is installed at an *N*‐arylsuccinimide rotor **14 a**, the rotation rate around the C−N axle can be accelerated upon the binding of an acetate guest (**14 a+OAc**, Scheme 14a).^[73]^ In the proposed mechanism, the acetate complex features a partially pyramidal imide carbonyl carbon which relieves the steric strain in the transition state.

Likewise, a difference in rotational speed is observed in **14 b** (R=H) and **14 c** (R=benzyl) depending on whether the phenol unit is protected or not. When phenol substituents are employed (**14 b**), a guest molecule can slow down the rotation speed of *N,N′*‐diarylnaphthalenediimides by two orders of magnitude.^[74]^ In this case, the diol has a low rotational barrier because of the intramolecular hydrogen bond with the imide carbonyl which stabilizes the planar transition state for the C−N rotations. Note that similar molecular design principles for acceleration of rotation are also found in other examples, including macrocyclic cyclophanes and N‐(substituted aryl)‐thiazoline‐2‐thione atropisomers.[^42b^, ^45^] In all cases addition of a guest such as acetic acid, acetone, and dimethylsulfoxide may disrupt the intramolecular hydrogen bonds and modulate the rotation rate according to their hydrogen‐bond‐accepting ability.

**Scheme 15:**
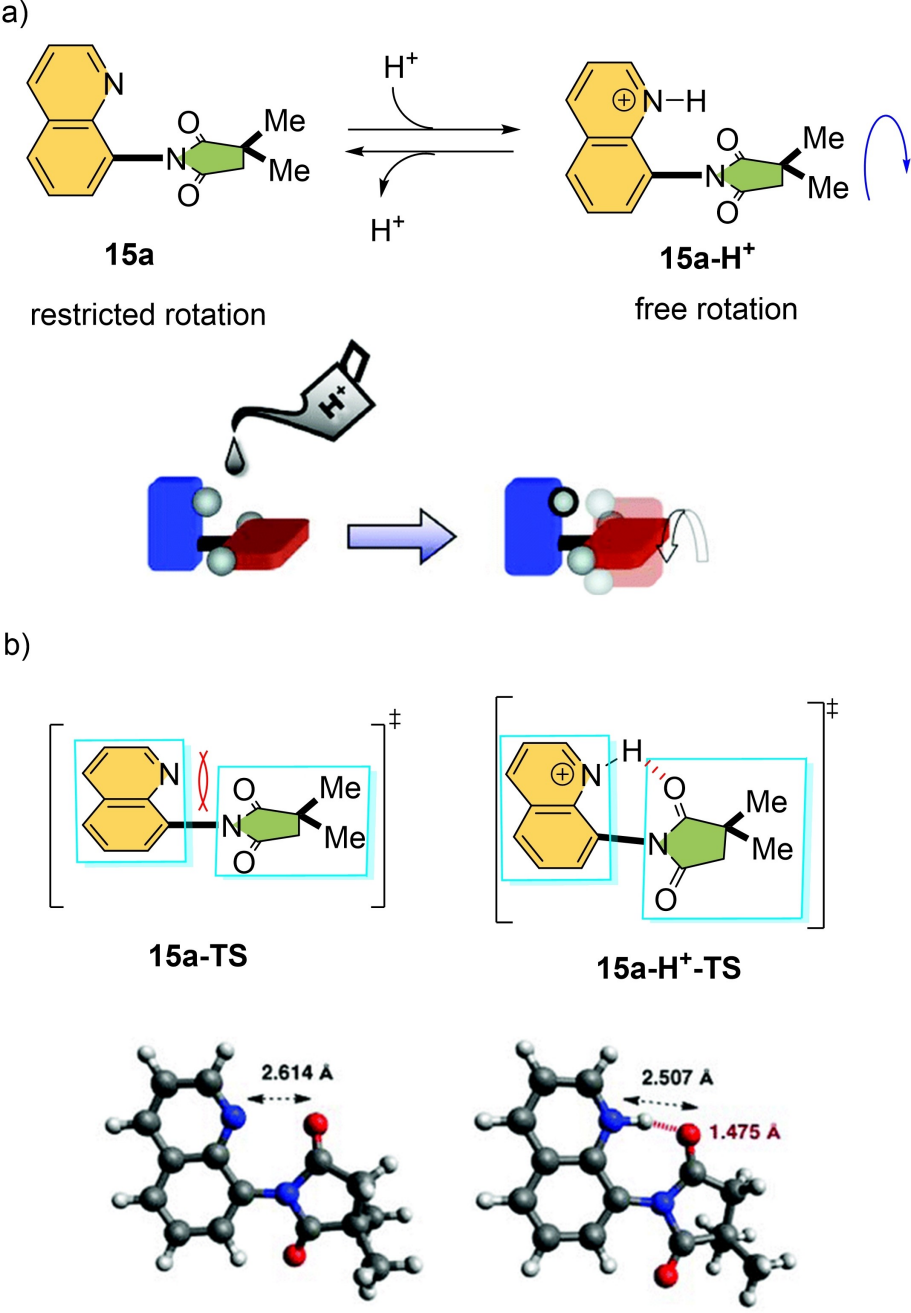
a) An acid‐accelerated molecular rotor b) H‐bonding interaction stabilizes the planar TS. Reproduced with permission from ref. [75] Copyright 2012, American Chemical Society.

Rotation around a C_aryl_−N_aryl_ single bond in N‐substituted imide **15 a** is restricted due to the lone‐pair–lone‐pair interaction between the imide carbonyl and the quinoline nitrogen. Based on this structure, proton‐responsive molecular rotor **15** was designed which rotates 10^7^ times faster upon addition of methanesulfonic acid (MeSO_3_H) as a stimulus (Scheme 15a).^[75]^ In this case, the planar transition state (**15 a‐H^+^‐TS**) is stabilized through an intramolecular H‐bond as shown in Scheme 15b, thereby, accelerating the rotation speed at the C−N axle from *t*_1/2_=24 min for the parent rotor (**15 a**) to *t*_1/2_=2×10^−4^ s for **15 a‐H^+^**.

**Scheme 16:**
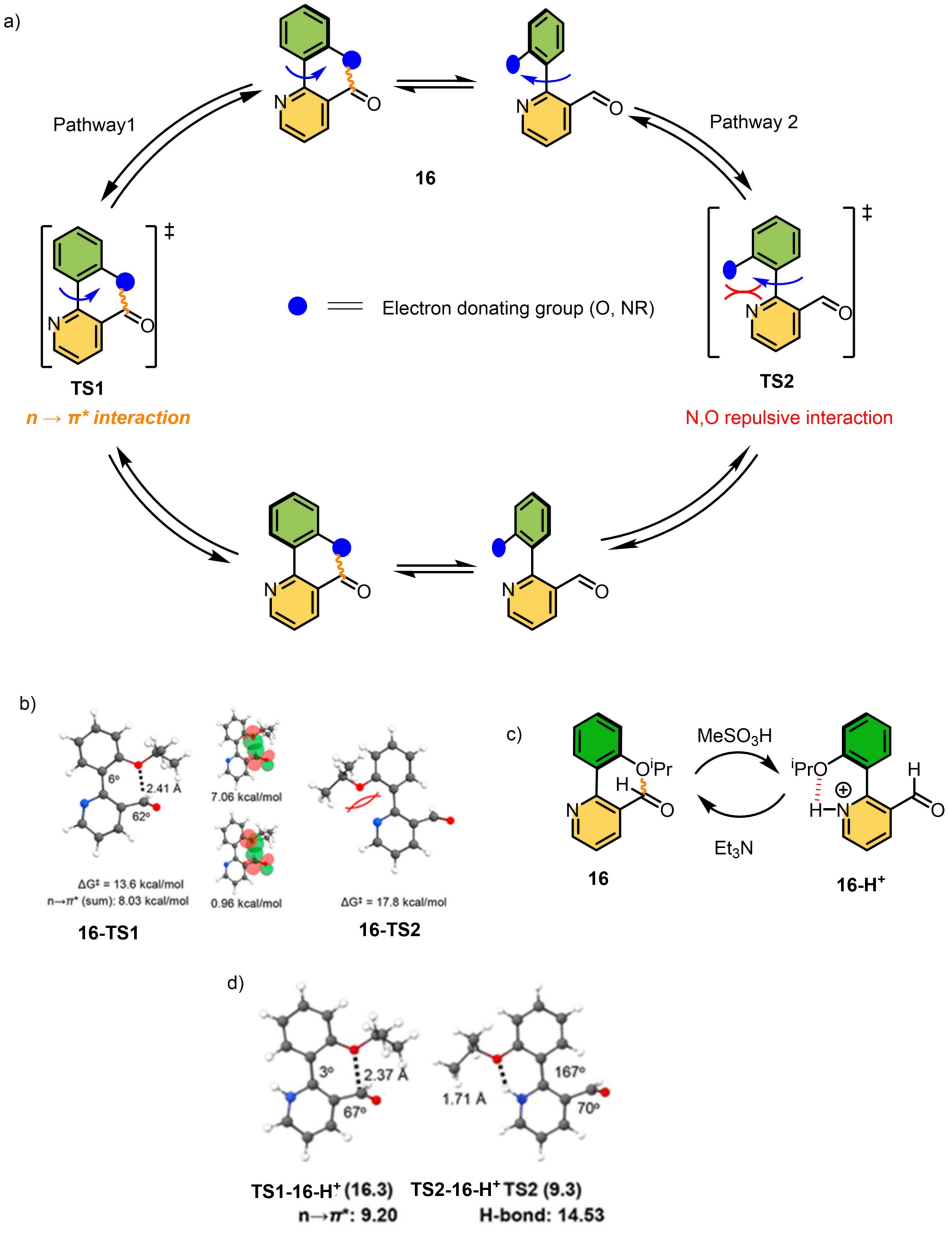
a) Rotational pathways of acid accelerated molecular rotor. b) DFT calculations of two different planar transition states of **16**. c) An acid acid/base‐mediated rotational switch. d) DFT calculations of two different planar transition state of **16‐H^+^**.^[76]^

More recently, another acid/base‐driven switchable molecular rotor was developed that can take a reverse rotational path by manipulating the n→π* interaction.^[76]^ The aldehyde **16**, which has two *ortho*‐substituents on opposite sides of the biaryl, can undergo free rotation around the C−C biaryl axle due to a low rotational barrier with Δ*G* ^≠^=12.6 kcal mol^−1^ (at 298 K in acetonitrile). This free rotation can follow two different pathways as shown in Scheme 16a. Because of the stabilization in the planar transition state **TS1** through a facile n→π* interaction compared to the N,O repulsive interaction (Scheme 16a), the rotation of rotor **16** likely occurs following Pathway 1 (where planar **16‐TS1** is stabilized by 4.2 kcal mol^−1^ compared to **16‐TS2**). Interestingly, the rotor rotation around the C−C single bond can be reversed by stabilizing the planar **TS2** in Pathway 2, using a chemical stimulus. The authors introduced another additional competing intramolecular interaction in the form of H‐bonding by protonating the pyridine nitrogen. In the protonated form of the rotor (**16‐H^+^**, Scheme 16c), **TS2‐16‐H^+^** (9.3 kcal mol^−1^) is stabilized over **TS1‐16‐H^+^** (16.3 kcal mol^−1^), thereby allowing rotation to take place following Pathway 2 (Scheme 16a). This is also accompanied by an acceleration of the rotation, as the barrier of interconversion is decreased to 10 kcal mol^−1^ due to this facile H‐bonding interaction.

**Scheme 17:**
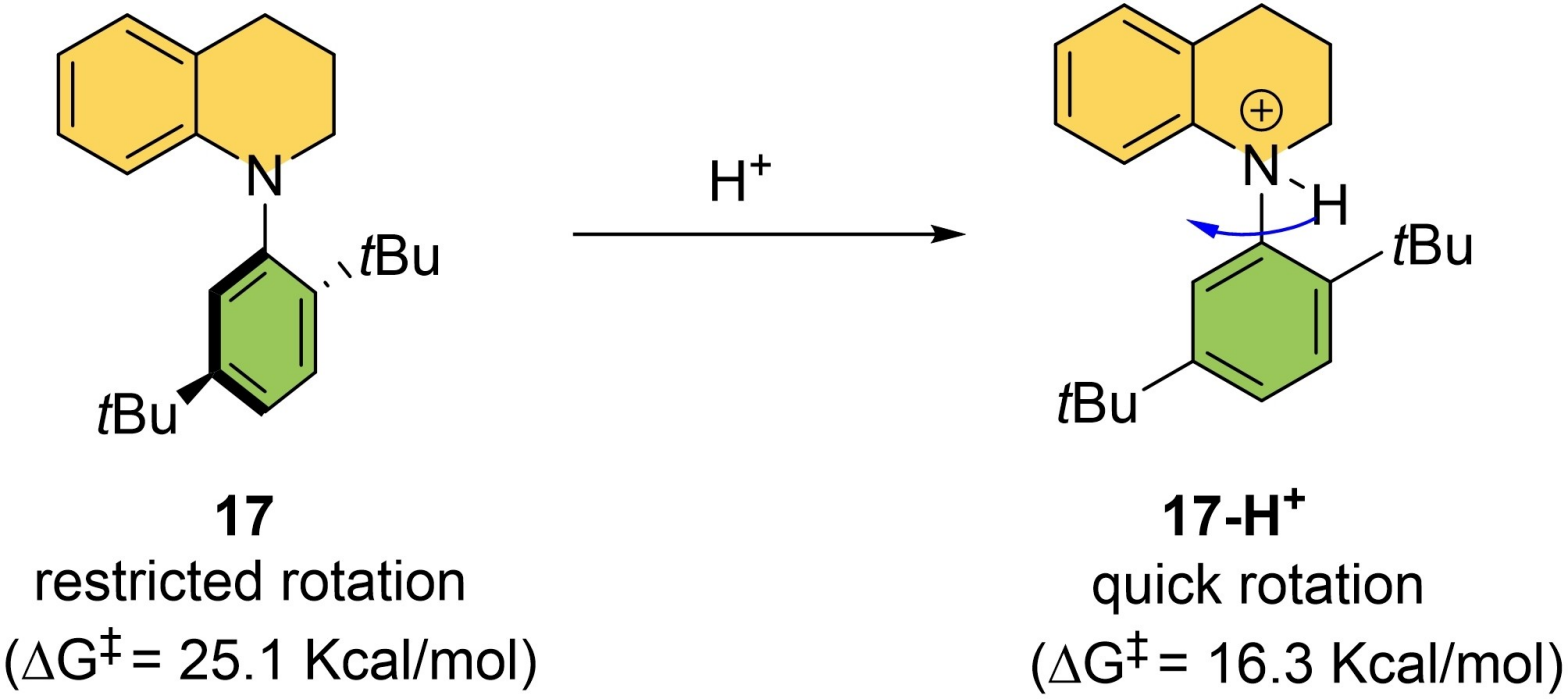
An acid‐accelerated molecular rotor.^[77]^

Kitagawa and co‐workers used a different strategy to develop an acid‐accelerated N‐substituted tetrahydroquinoline‐based molecular rotor (which displays axial chirality) shown in Scheme 17.^[77]^ Rotor **17** exists as a stable atropisomer at room temperature with a barrier of rotation Δ*G* ^≠^=25.1 kcal mol^−1^. Upon protonation of the nitrogen center with an acid, the barrier of the rotation around the C−N single bond is dramatically lowered to Δ*G* ^≠^=16.3 kcal mol^−1^ and racemization is complete in 10 min. A similar behavior was observed in acyclic diarylamines by the Clayden group.^[78]^ This dynamic behavior is attributed to the pyramidalization of the nitrogen center involved in the restricted bond rotation. Upon protonation, the conversion from sp^2^ to sp^3^ decreases the steric demands around the nitrogen atom, increasing the freedom for C−N bond rotation.

**Scheme 18:**
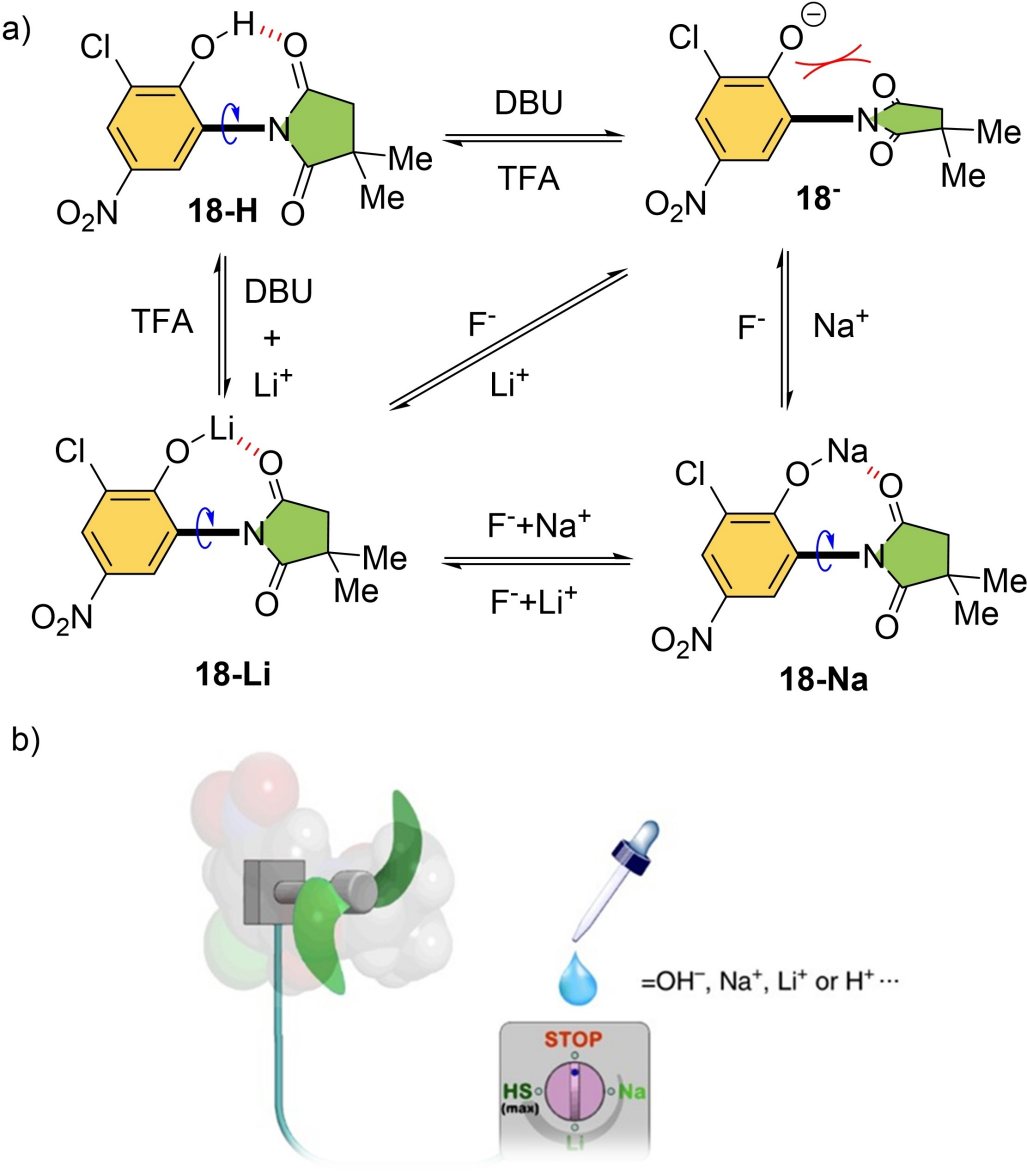
a) Succinimide molecular rotor with tunable rotation speed via chemical stimuli. b) An illustration of the molecular rotor which can mimic a macroscopic electric fan.^[79]^

In 2018, the group of Wang demonstrated modulation of rotational speed of a related succinimide molecular rotor around its C−N bond in response to external metal cations (**18** in Scheme 18).^[79]^ The authors combined H‐bonding, electrostatic repulsion, and metal‐coordination interactions in their designed rotor, and controlled the degrees of the various interactions by using multiple external stimuli such as acid/base and metal cations. Rotor **18‐H** has a high rotation speed (10^4^ Hz at 298 K) at the original state.

When deprotonated at the phenol moiety, the rotation becomes restricted because of the intramolecular electrostatic repulsion between the phenolate anion and the carbonyl groups in **18** ^−^. However, addition of alkali metal cations (Li^+^ or Na^+^) introduces an electrostatic bridge between the stator and the rotator and lowers the energy barrier for rotation. This acceleration is dependent on the radius of the metal cation (10^−1^ Hz for **18‐Na**, 10^0^ Hz for **18‐Li**). This results in multiple stages for the rotational speed, mimicking a macroscopic fan (Scheme 18b).

**Scheme 19:**
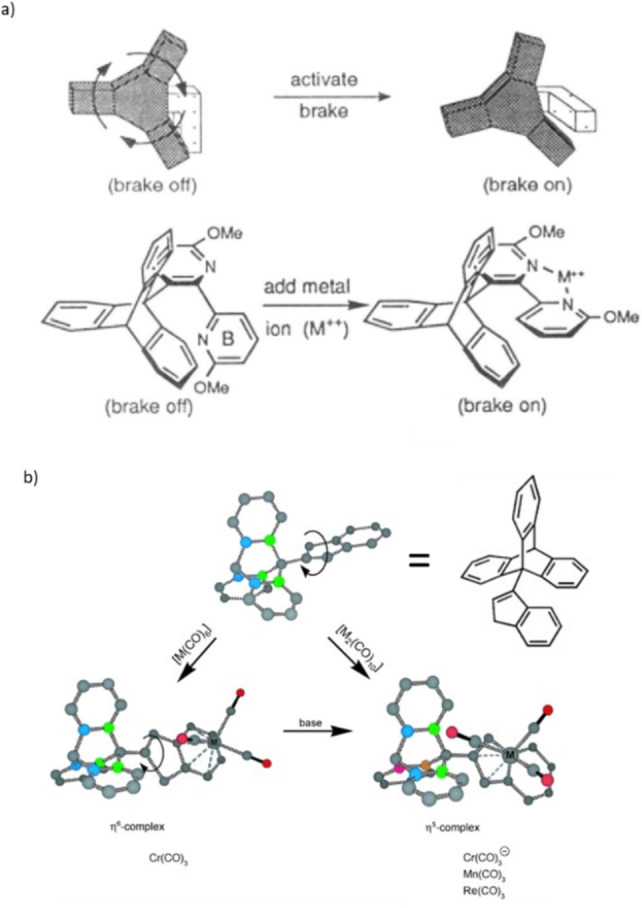
a) Kelly's molecular brake regulated by metal coordination. b) Paddlewheel rotor with indenyl‐metal complex. Reproduced with permission from ref. [80] Copyright 1994, American Chemical Society, and ref. [81b] Copyright 2012, American Chemical Society.

The dynamic nature of coordination bonds (formation and dissociation) can directly facilitate molecular switching. The first example of a rotor controlled by metal coordination is Kelly's molecular brake, reported back in 1994 (Scheme 19).^[80]^ The rotor consists of a triptycene rotator and a bipyridine metal‐binding unit that are connected though a C−C single‐bond axle. An intramolecular structural and conformational change triggered by metal coordination results in stopping the rotation of a triptycene rotor. By forcing the bipyridyl unit into a rigid, planar conformation upon coordination, steric clash between the brake and the triptycene unit increases the barrier to rotation of the rotor. Inspired by this triptycene‐based motif, an indenyl‐metal complex (Cr, Mn or Re) directly attached to the paddlewheel was designed to regulate the rotation. Using base as a chemical stimulus, a haptotropic shift was triggered, moving the organometallic core from the far six‐membered ring of the indane moiety (*h* ^6^) to the five‐membered ring (*h* ^5^) located close to the triptycene. This structural change slowed down the rotation speed of the triptycene by ca. 10^8^ times.^[81]^

In the previous sections, we have shown several examples of chemically induced geometrical molecular switches and acceleration/deceleration of the rotation of C−C and C−N single bonds of molecular rotors. Some of these concepts were key to the development of chemically triggered unidirectional motors.

However, these intriguing small molecular rotors and switches do not rotate unidirectionally because there is no directional constraint, and therefore they cannot be called molecular rotatory motors. In the following section, we will discuss various key principles required for the development of a new class of molecular motors and provide examples and the state of the art in the development of chemically driven biaryl‐based rotatory motors.

## 4 Design and Function of Biaryl‐Based Molecular Motors

In this section, the design of molecular rotatory motors and their detailed mechanistic and stereochemical properties will be presented. The main discussion will focus on C_aryl_−C_aryl_ bond rotation in biaryls and the various approaches are illustrated. For a simple unsubstituted biphenyl, rotation around the axle is often considered to be “free”, i.e., the energy barrier is less than the thermal energy at room temperature.^[28]^ However, when the *ortho* positions of the phenyl ring are substituted with functional groups, the barrier of rotation becomes high enough to isolate individual conformers, which are called “atropisomers”.^[82]^

**Figure 2:**
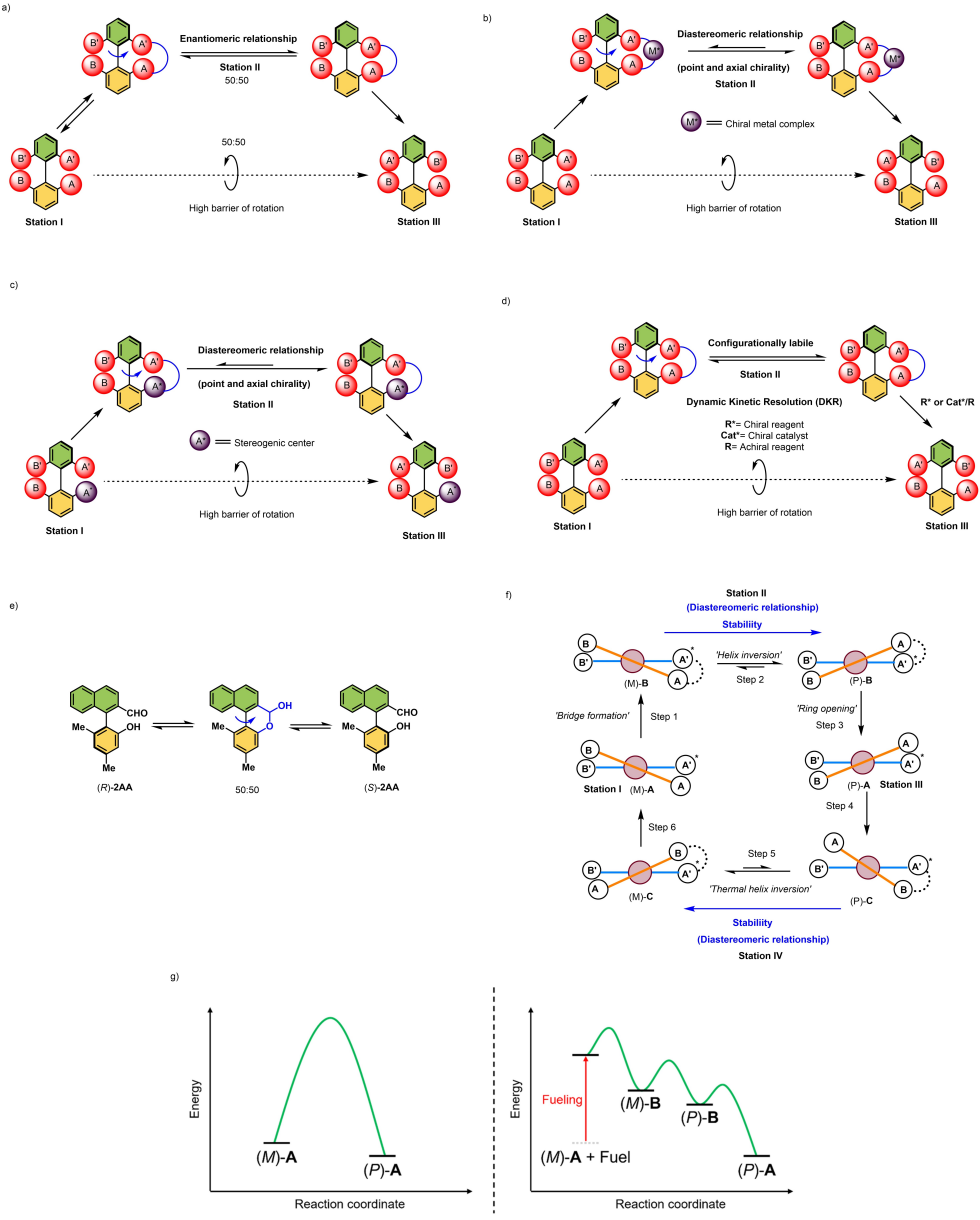
a) Proposed mechanism for the atropisomerization of a configurationally stable biaryl compound; b–d) a directed facile atropisomerization of the bridged biaryl; e) Proposed mechanism for the racemization of (*R*)‐**2 AA**; f) Schematic representation of a biaryl molecular motor. g) Left: Energy profile diagram of the atropisomerization process from (*M*)‐**A** to (*P*)‐**A**, where the barrier of rotation without chemical fuel is high; right: the energy profile of unidirectional rotation from (*M*)‐**A** to (*P*)‐**A** using a chemical fuel.

In Figure 2, if A≠B and A′≠B′, stereoisomers at **Station I** and **III** are non‐superimposable and display axial chirality (Figure 2a–d). The energy required for the interconversion between enantiomers at **Station I** and **III** (atropisomerization) primarily depends on the size and the number of substituents at the *ortho*‐positions as mentioned earlier. In the case of biaryls with multiple substituents, rotation is restricted but atropisomerization can be achieved under certain conditions, i.e., “nondirectional” rotation of one of the aryl rings. One such process is thermal isomerization. Upon heating, biaryls can racemize via a sterically preferred nonplanar transition state where the *ortho* substituents can pass each other.[^82^, ^83^] Using quantum‐chemical calculations, it has been shown that thermal rotation about a biaryl axis occurs in twisted (i.e. nonplanar) transition states in which the *ortho* substituents and the aryl rings are distorted, permitting them to pass more easily than in a rigid planar transition state.^[84]^

Alternatively, bridging the *ortho*‐substituents can reduce the barrier of atropisomerization, as demonstrated by Bringmann and co‐workers.^[85]^ This is because when a covalent bridge is formed between the *ortho*‐substituents of the lower and upper aryl rings, the angle between the planes of the two benzene rings of the biaryl moiety is reduced to about 30–40°, which lowers the activation barrier for helix inversion to less than 20 kcal mol^−1^.^[86]^ The same principle has subsequently been used to develop many elegant asymmetric approaches for the synthesis of axially chiral enantioenriched biaryls.[^17a^, ^87^] In a similar fashion, bridges based on noncovalent interactions, such as those formed by hydrogen bonds or metal coordination between *ortho*‐substituents attached to biphenyl groups, can also enable helix inversion. An elegant example of such isomerization process is the slow racemization of hydroxy aldehyde compound **(*R*)‐2 AA** (Figure 2e), a fourfold substituted biaryl that should be rotationally stable based on the result of theoretical calculations. But in reality, **(*R*)‐2 AA** racemizes easily at room temperature, presumably because of the formation of a six‐membered lactol intermediate as shown in Figure 2e. As a consequence, steric interactions between the *ortho*‐substituents are greatly altered and a lower rotational barrier is achieved. Inspired by this observation, introducing a chemical bridge between *ortho*‐substituents can be a definite motif for promoting atropisomerization, i.e., 180° rotation of upper aryl ring (Figure 2a). Therefore, in order to move from **Station I** to **Station III**, atropisomer at **Station I** can be converted to a bridged intermediate (**Station II**) by replacing the *ortho*‐substituents with a single bridging unit (Figure 2a). Then the atropisomers at **Station II** will be configurationally labile and helix inversion is ideally possible.^[30]^ Subsequently, a ring‐opening reaction of the bridging unit at **Station II** would “lock” the conformation, leading to the formation of a stable atropisomer at **Station III** and completing the 180° rotation. However, using this approach, full unidirectionality cannot be obtained because half of the bridged intermediate (**Station II**) returns to **Station I**, owing to the same ground state energy of the enantiomers. To ensure directionality, it is postulated that the introduction of additional (point) chirality besides the axial chirality is necessary in the bridging **Station II**. The combination of two different chiral moieties makes atropisomers at the **Station II** diastereomeric in nature. As a result, this creates sufficient stereochemical bias and one form of the helical intermediates becomes thermodynamically more preferred over the other. This could possibly result in complete directional transformation from **Station I** to **Station III** after a ring‐opening reaction. The following three strategies can be used to achieve full atropo‐diastereomerization via a facile chirality transfer from a stereogenic center to the rotational axle.

1. As shown in Figure 2b, the stereochemical information can be transferred by introduction of a chiral ligand or a chiral metal complex (M*) directly to the bridged intermediate in **Station II**. This external chiral information ultimately governs the direction of rotation to form **Station III**.
2. The presence of “point chirality” (A*) in the close vicinity of the axle (preferably at one of the *ortho* positions) can also promote a directed rotation from **Station I** to **Station III**, as shown in Figure 2c. Again, the presence of two stereochemical elements, fixed stereogenic chirality at A*center and dynamic axial chirality in the biaryl, makes the complexes in **Station II** diastereomeric in nature and control of directionality is expected.
3. Figure 2d illustrates the final strategy, in which a directional bias can be achieved by converting bridged biaryls at **Station II** directly to an axially chiral biaryl at **Station III** via an atropo‐enantioselective ring‐opening reaction, utilizing a chiral reagent or the combination of chiral catalyst and achiral reagent. Contrary to the strategy shown in Figure 2a, chiral nucleophiles or a chiral catalyst (e.g., an organocatalyst) is used to achieve a dynamic kinetic resolution (DKR), and two enantiomers at **Station II** are converted to a single atropisomer by a stereoselective ring‐opening. It should be noted that the chirality‐matched reagent or catalyst itself determine the extent of conversion from **Station I** to **Station III**.

Designing a chemically driven molecular motor has been one of the long‐standing goals of many research groups. (For other approaches based on mechanically interlocked systems, see refs. [31, 88] and for chemically driven propulsion systems, see ref. [89].) In Figure 2f, a schematic illustration of a unidirectional biaryl motor cycle is provided, where the rotator rotates with respect to the stator in an anticlockwise fashion. A 360° rotational cycle consists of six distinctive steps. Note there is hindered biaryl rotation due to the presence of substituents (A, A′, B, and B′) but conformational change is possible e.g. bridging A′, B (Step 4) or A, A′ (Step 6). The stepwise chemical reactions through steps 1, 2, and 3 (step 2 being the switching step) affords the first half of the unidirectional rotatory cycle (180° rotation, half‐turn), where A crosses A′ and B passes B′ and similarly steps 4, 5, and 6 (where step 5 is the switching step) are responsible for the second half of the rotary cycle (360° rotation, full turn), where B passes A′ and A crosses B′. Step 1 involves the formation of a configurationally unstable diastereomeric intermediate (*M*)‐**B** by selectively connecting A and A′ through a chemical process, lowering the biaryl rotational barrier. Then at Step 2, (*M*)‐**B** undergoes a thermal helix inversion to a stable isomer (*P*)‐**B**. Diastereoisomerization is responsible for this directional switching. In the bridge form, the biaryl stereogenic centers can significantly affect the atropo‐diastereomeric equilibrium. When the bridge switches from (*M*)‐**B** to (*P*)‐**B** (which are diastereomers), it favorably adopts a thermodynamically more stable conformation. Selective bond cleavage at Step 3 results in a half turn of the motor, forming (*P*)‐**A** at **Station III**. In a similar fashion, at Step 4, the selective bond formation of A′ and B delivers another configurationally labile bridged intermediate (*P*)‐**C**, and the second thermal helix inversion takes place. By cleaving the bridge at Step 6, the motor returns back to its original form at **Station I** and a full 360° rotation is accomplished.

This chemically driven unidirectional rotation process can also be explained by the concept of kinetic asymmetry.^[90]^ The energy diagram of fueled 180° unidirectional rotation is depicted in Figure 2g. As demonstrated in Figure 2g, the activation barrier for the atropisomerization process, i.e., the process of converting (*M*)‐**A** to (*P*)‐**A** without using fuel, is relatively high (see the left panel in Figure 2g). Figure 2g (right panel) shows that after the fuel supplies chemical energy, the system reaches a higher energy state, and a bond between the upper and the lower halves is made to form a bridged compound (*M*)‐**B**. By the subsequent helix inversion, thermodynamically preferred isomer (*P*)‐**B** is obtained. The unidirectional 180° rotation is completed by the subsequent ring‐opening step. In a similar mechanism the second rotational cycle is accomplished to complete 360° rotation.

## 5 Directed Rotation by Formation of a Chemical Bond

**Scheme 20:**
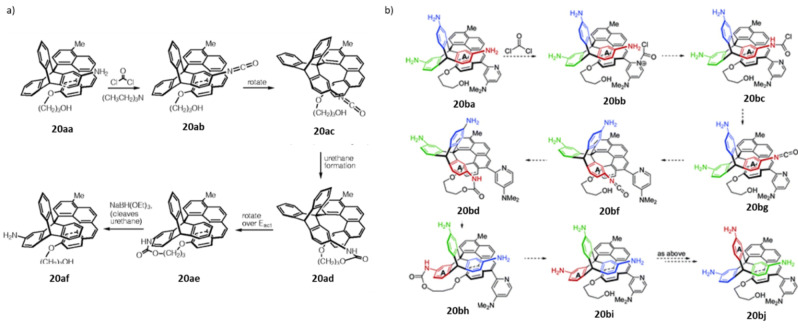
a) Unidirectional 120° rotation in a three‐bladed triptycene unit using phosgene as “fuel”. Reproduced with permission from ref. [91] Copyright 1999, Springer Nature. b) A schematic representation of a possible rotatory molecular motor by Kelly and co‐workers. Reproduced with permission from ref. [92] Copyright 2007, American Chemical Society.

In this section, we discuss the early designs of molecular motors, which eventually emerge as unidirectional switches, but failed to fulfill the criteria of a molecular motor according to definition discussed in the introduction section. In 1999, Kelly and co‐workers were the first to observe a unidirectional rotation around a single bond by converting chemical energy into rotational energy.^[91]^ Their molecular design is made up of a three‐bladed triptycene unit (having an amine functional group) as a rotator and a helicene stator possessing an aliphatic alcohol as shown in Scheme 20a. In the first step of their rotational cycle, upon addition of phosgene as “fuel” under basic conditions, the corresponding amine group is converted to isocyanate **20 ab**. Then, the inherent thermal rotation (conformational changes) brings the triptycene moiety close to the propyl alcohol attached to the helicene unit, giving a metastable urethane **20 ad**. We hypothesized that the rotation in **20 ab** is possible due to the linear structure of the isocyanate unit in contrast to the pyramidal amine unit in **20 aa**. Compound **20 ad** is then converted into the thermodynamically more stable product, **20 ae**, resulting in a 120° rotation via an irreversible rotation around the interconnecting C−C bond by ring strain relief. The subsequent cleavage of the urethane bond leads to **20 af**, a rotamer of **20 aa**, which cannot return to its original state. Nevertheless, this research represents an important milestone in the early development of a unidirectional rotation about a single bond, and set the stage for the future improved designs.

With the aim of developing a functional rotatory motor, a few changes were made to the initial design (Scheme 20b). 1) An amino group was installed on each blade of the triptycene rotator unit; and 2) a DMAP (4‐dimethylaminopyridine) directing group was attached to the helicene stator (**20 ba**), with the hope that it would selectively deliver the carbonyl chloride moiety to its closest amino group.^[92]^ If subsequent events follow accordingly as shown in Scheme 20b, then a repetitive unidirectional rotation would be possible. Having demonstrated the phosgene‐mediated 120° unidirectional rotation in their previous motif, the authors sought to investigate the rotational cycle starting from **20 ba**. The amine closest to the DMAP unit was expected to react with the *N*‐chlorocarbonylpyridium in **20 bb**. However, the addition of phosgene to **20 ba** led to an intermolecular urea formation, which gives rise to an undesired polymer formation. A number of further experiments were conducted to suppress the polymerization. Although, selective formation of **20 bg** was possible, the formation of the corresponding urethane **20 bd** was found to be extremely challenging. This suggests that the rotation around the triptycene/helicene axle is restricted due steric interactions between the added functional groups.

**Scheme 21:**
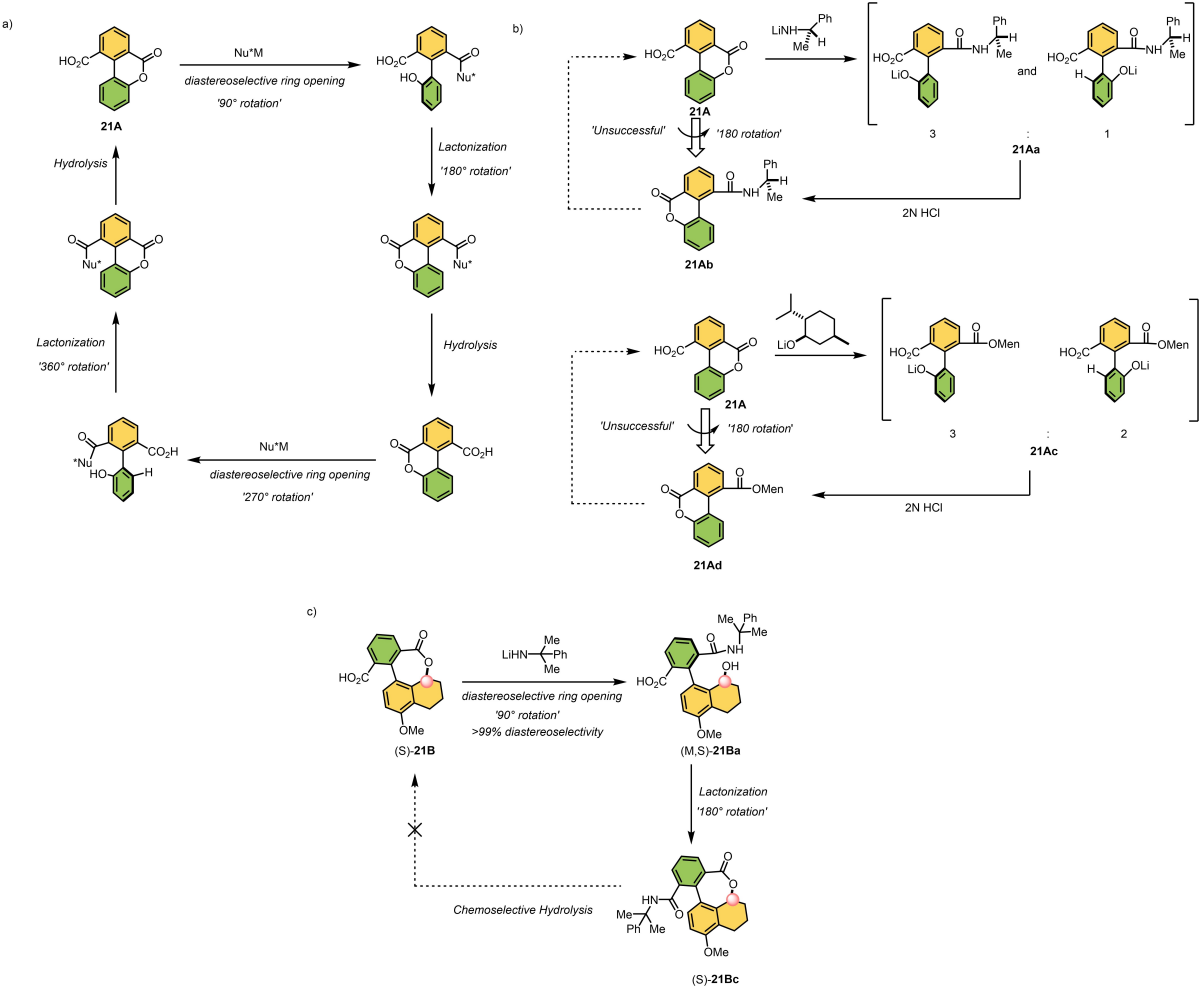
a) A blueprint towards the unidirectional bond rotation around the biaryl single bond proposed by Branchaud and co‐workers. b) Demonstration of a unidirectional 180° rotation using chiral nucleophiles. c) Rotational behavior of lactone (*S*)‐**21 B** bearing an extra asymmetric center. Note: All the steps shown is performed using a racemic mixture, but only the rotational pathway of (*S*)‐21B is shown for clarity.^[93]^

Subsequently, Branchaud and co‐workers envisioned two different concepts towards a molecular motor based on a chirality‐directed bond rotation in a biaryl system.^[93]^ The key steps in their strategies are a lactonization reaction and a selective ring opening with an external nucleophile as shown in Scheme 21. In their first design, the authors hypothesized that with the help of a chiral nucleophile, a net directed 90° rotation of the upper “arene” ring could be achieved via a diastereoselective ring opening of cyclic lactone **21 A** (Scheme 21a). Subsequently, if the removal of the nucleophile and reformation of the lactone unit happened in succession without biaryl atropisomerization, a functional motor could be obtained. To realize their rotational cycle, the authors first attempted two different ring‐opening reactions of lactone **21 A** with two chiral nucleophiles (Scheme 21b).^[93a]^ First, lactone **21 A** was opened diastereoselectively using a chiral lithium amide, giving a diastereomeric mixture of both (*P*)‐**21 Aa** and (*M*)‐**21 Aa** in a 3 : 1 ratio. Similarly, using lithium menthoxide as a nucleophile, (*P*)‐**21 Ac** and (*M*)‐**21 Ac** could be obtained in a ratio of 3 : 2. In a subsequent recyclization reaction of the corresponding phenolates with the unprotected carboxylic acid on the opposite side of the aryl ring, the two lactones, **21 Ab** and **21 Ad**, were formed. As a result, a unidirectional 180° rotation was obtained in both cases. One should note that only a partial directionality is achieved as the diastereoselectivity of the ring‐opening steps were rather poor. Although it is a promising half‐turn, the second half‐turn could not be completed as the hydrolysis of both amide **21 Ab** and ester **21 Ad** was found to be unselective.

Similarly, the authors modified their initial design by introducing an extra stereogenic center at the benzylic position next to the biaryl axle in (*S*)‐**21 B** (Scheme 21b).^[93b]^ In the anticipated design, the net directed rotation could be accomplished with the help of an extra stereogenic center close to the axle. (Note that all the steps shown in Scheme 21 were performed using a racemic mixture, but only the rotational pathway of (*S*)‐**21 B** is shown for clarity.) First, the cyclic lactone (*S*)‐**21 B** is subjected to a ring‐opening reaction to form the corresponding amide (*M,S*)‐**21 Ba**. The ring opening proceeds with excellent diastereoselectivity (>99 %) towards (*M,S*)‐**21 Ba** as compared to their previous approach, showing the importance of the additional stereogenic center in the benzylic position of **21 B**. Immediate relactonization of the hydroxyl unit with the unprotected carboxylic acid resulted in 180° unidirectional rotation, delivering (*M,S*)‐**21 Bc**. Again, chemoselective hydrolysis of the amide unit was unsuccessful and full unidirectional rotation was not reached. We note that to prove a complete unidirectionality for their first 180° rotational cycle follow‐up experiments need to be performed with an enantiomerically pure **21 B**.

### 5.1 Molecular Motors Driven by Chemical Energy

**Scheme 22:**
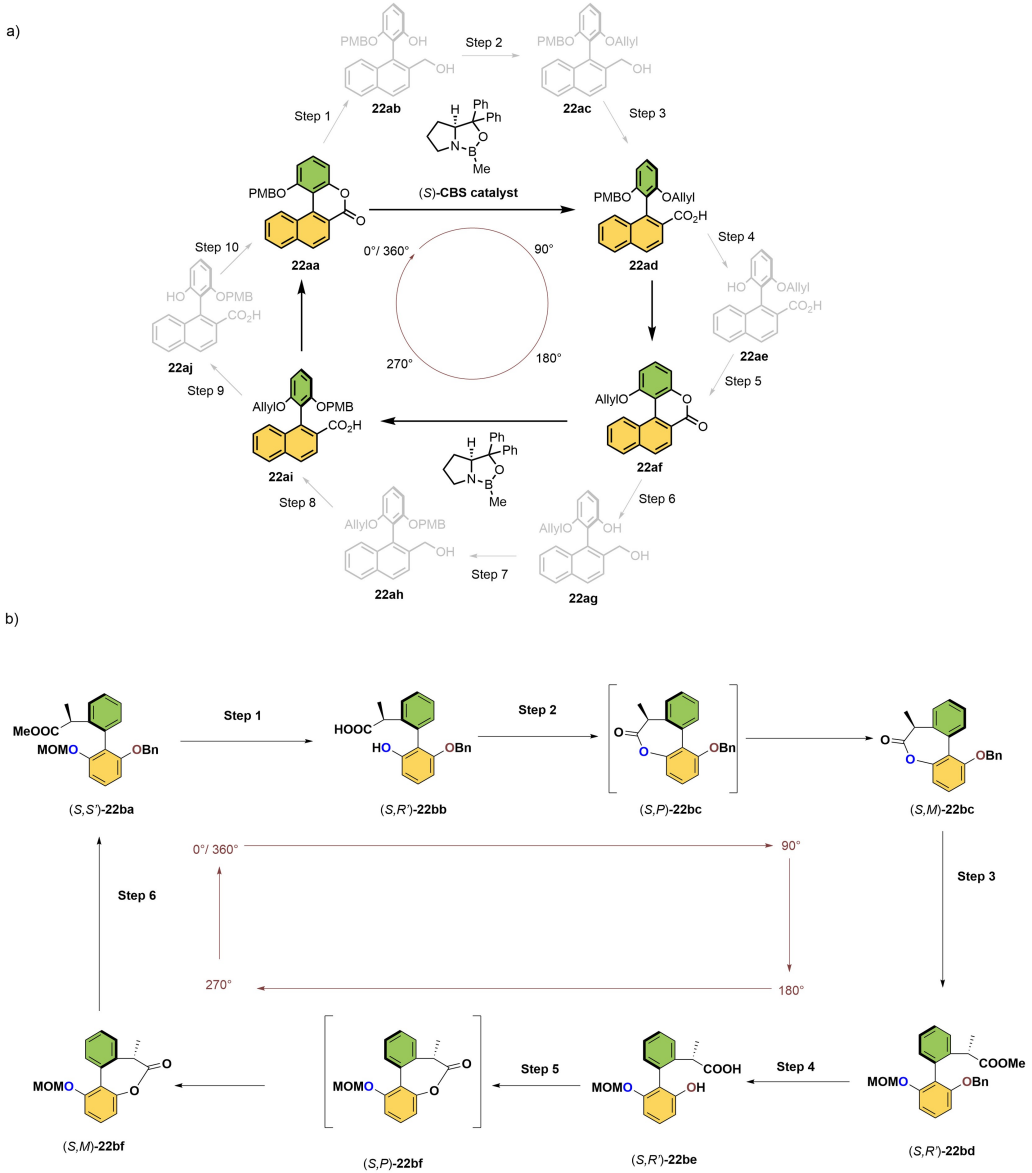
a) First example of a chemically driven rotary molecular motor by Feringa and co‐workers. Step 1: Enantioselective ring opening of cyclic lactone using (*S*)‐2‐methyl‐CBS‐oxazaborolidine solution then BH_3_; Step 2: Protection with allyl bromide; Step 3: Oxidation of benzyl alcohol using CrO_3_.H_2_SO_4_.H_2_O,then NaClO_2_; Step 4: Removal of PMB using Ce(OTf)_3_; Step 6: Another enantioselective ring opening of cyclic lactone using (*S*)‐2‐methyl‐CBS‐oxazaborolidine solution then BH_3_; Step 7: Protection with *p*‐methoxybenzyl chloride; Step 8: Oxidation of benzyl alcohol with MnO_2_ then NaClO_2_; Step 9: Removal of allyl ether with Pd(PPh_3_)_4_/HCO_2_H.^[94]^ b) The 360° rotation of a molecular motor via chiral bridged lactone formation by Feringa and Zhao. Step 1: Hydrolysis using HCl/MeOH; Step 2: Lactonization using EDCI; Step 3: Diastereoselective ring opening using MeONa and protection of phenolic hydroxyl group with MOMCl; Step 4: Removal of the benzyl protecting group using Pd/C, H_2_; Step 5: Lactonization using EDCI; Step 6: Diastereoselective ring opening using MeONa and protection of phenolic hydroxyl group with benzyl bromide.^[95]^

In this section we discuss genuine molecular motors that can undergo repetitive unidirectional rotation in response to specific chemical stimuli. Building on stereodynamic principles discussed in the previous section,^[93]^ our group reported the first example of a fully functional unidirectional, chemically driven rotatory molecular motor based on asymmetric catalysis as the key step to achieve directional control. The full rotational cycle also takes advantage of orthogonal phenolic protecting groups. The 180° rotation, based on asymmetric catalysis as a key step, begins with a bridged lactone **22 aa** (Scheme 22a).^[94]^ In this state, due to the presence of the bridging unit, aryl rings can undergo enantiomeric interconversion, i.e., rotation around the motor axle to give a pair of enantiomers. As shown in Step 1, an enantioselective reduction (with an enantiomeric ratio of 97 : 3) opens the ring of the cyclic lactone **22 aa** with the help of a chiral CBS catalyst ((*S*)‐2‐methyl‐oxazaborolidine). The corresponding acid **22 ad** is then obtained by O‐protection with an allyl group (Step 2) and oxidation of the benzylic alcohol (Step 3), resulting in a net clockwise 90° rotation. Subsequently, selective removal of a benzylic ether (Step 4) provides again a cyclic bridged lactone, **22 af**, via the lactonization of **22 ae** (Step 5). The combination of these steps corresponds to a unidirectional half‐turn of the molecular motor. In a similar process, a sequence of catalytic asymmetric ring opening (Step 6), reprotection of benzyl ether (Step 7), and oxidation of the benzylic alcohol (Step 8), gives the enantiomer **22 ai** as a result of directed 270° rotation from **22 aa**. Finally, removal of the allyl protecting group (Step 9) and spontaneous lactonization regenerate the initial bridged lactone **22 aa**, which completes a full 360° rotatory cycle. This net directionality of the rotor is solely dependent on the point chirality present in the CBS catalyst, meaning that the directionality can be inverted using the other enantiomer of the CBS catalyst. Although a proof of principle of 360° unidirectional rotation around a biaryl single bond is achieved, it should be noted that one cycle of rotation in this system goes through ten consecutive steps with >90 % unidirectionality. Recently, the groups of Zhao and Feringa have improved the directionality of rotation with a modified biaryl motif (Scheme 22b),^[95]^ based on combination of axial and central chirality to control diastereoselectivity. The number of steps for a full rotatory cycle is reduced to six and stereochemical directionality exceeded 99 %. A stereogenic center is introduced at the benzylic position of (*S,S′*)‐**22 ba** in order to direct the motor rotation (Figure 2c). This design contrasts with the use of the external chiral catalyst as shown in our previous approach featuring enantioselective transformation to control directionality. Again two stereochemical elements are present, i.e., central and axial chirality, necessary to bias rotation by diastereoselective transformation. The motor cycle begins with both deprotection of methoxymethyl group (MOM) and hydrolysis of the ester group of (*S,S′*)**‐22 ba** (Step 1), after which an intramolecular esterification (lactonization) leads to the formation of a metastable cyclic lactone (*S,P*)**‐22 bc** (Step 2). The newly formed (*S,P*)**‐22 bc** is in equilibrium with diastereomer (*S*,*M*)**‐22 bc**, which is thermodynamically more stable, and a thermal helix inversion of the lactone proceeds at this step. Then, diastereoselective ring opening of lactone (*S*,*M*)‐**22 bc** with sodium methoxide (Step 3) and subsequent reprotection of the phenolic hydroxyl group results in a 180° rotation of the upper phenyl ring containing the carboxylic ester group. The second half‐turn is achieved by the removal of the benzyl group and lactonization using EDCl (1‐ethyl‐3‐(3‐dimethylaminopropyl)carbodiimide, Steps 4 and 5). The resulting bridged lactone, (*S,P*)**‐22 bf**, can now undergo another thermal helix inversion to the stable atropisomer (*S,M*)**‐22 bf**. In a similar fashion, combination of a ring‐opening reaction of (*S,M*)**‐22 bf** and reinstallation of the benzyl protecting group delivers the starting isomer (*S,S*)‐**22 ba** (Step 6). This molecular rotary motor works in six individual steps and is the first example to show nearly complete unidirectionality (>99 %).

**Scheme 23:**
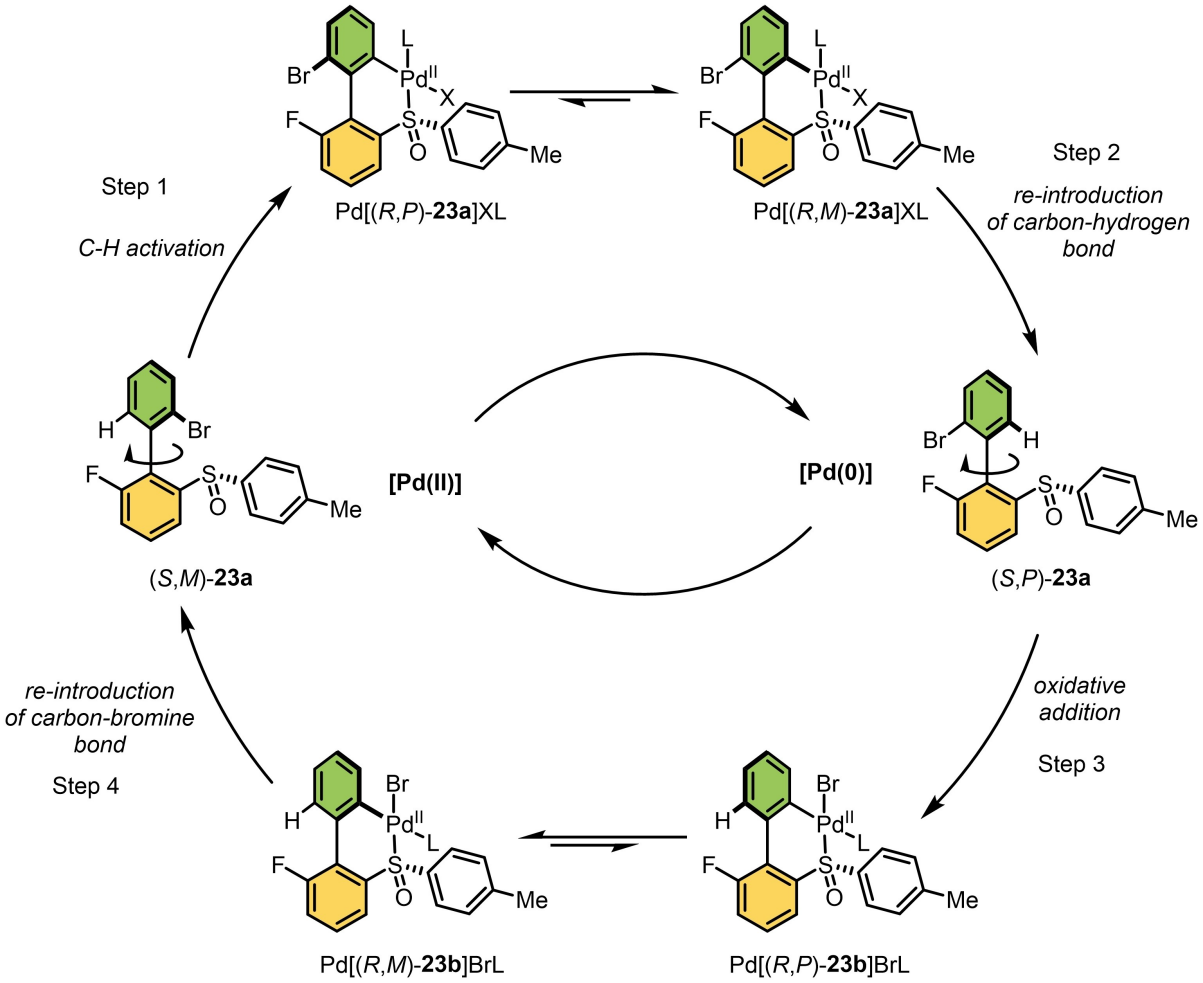
Palladium redox cycle driven molecular motor developed by Feringa and co‐workers. One full rotational cycle in four steps, Step 1: C−H activation (using Pd(OAc)_2_), Step 2: Reintroduction of C−H bond (using NaBH(OAc)_3_); Step 3: Oxidative addition (using Pd_2_(dba)_3_), Step 4: Reintroduction of C−Br bond (using NBS).^[96]^

The next challenge in this field is to develop an autonomous molecular motor that does not require sequential addition of chemical fuels and that has the properties of a catalytic motor. Towards this goal, our group designed a molecular motor driven by switching of the oxidation states of a Pd catalyst (Scheme 23).^[96]^ It should be noted that Pd has a dual role, acting as bridging unit to lower the barrier for the biaryl interconversion and as an active metal center for the C−H and C−Br bond activation. The cycle starts from a stable (*S,M*)‐**23 a** which does not undergo atropisomerization due to the high barrier of rotation (Δ*G* ^≠^=37.0 kcal mol^−1^). The starting material can be however converted to thermodynamically stable six‐membered cyclic palladacycle Pd[(*R,P*)‐**23 a**]XL by a C−H activation^[97]^ reaction assisted by sulfoxide as a directing group and a Pd^II^ catalyst.^[98]^ The presence of a sulfoxide stereogenic center in the six‐membered bridged palladacyle guides a thermal helix inversion to the more stable diastereoisomer Pd[(*R,M*)‐**23 a**]XL, according to the principle shown in Figure 2c. A subsequent proto‐depalladation^[99]^ reaction provides (*S,P*)‐**23 a**, leading to a net clockwise 180° rotation. By switching the oxidation state of the Pd source from (II) to (0), the catalyst selectively undergoes addition to the C−Br bond and forms oxidative addition complex Pd[(*R,P*)‐**23 b**]BrL. Next, another subsequent thermal helix inversion to the more stable diastereomer Pd[(*R,M*)‐**23 b**]BrL proceeds. Reintroduction of bromide using *N*‐bromosuccinimide (NBS) “locks” the conformation to give the starting isomer (*S,M*)‐**23 a**, resulting in a directed net 360° rotation. It was also demonstrated that Pd^0^ can be directly regenerated from the remaining Pd^II^ reagent after the first half‐turn by using an excess amount of tricyclohexylphosphine.^[100]^ Taking advantage of the Pd^0^/Pd^II^ redox cycle in combination with selective C−H and C−Br bond activation is an attractive approach towards the development of fully autonomous chemically powered molecular motor. In this study, the full 360° rotation cycle completes with an overall yield of 19 % over five steps. Clearly, there is still room for further improvement of the catalytic sequence, possibly by changing the directing group or the transformation methods of the catalyzed steps. This concept might be extended further towards an electric motor by the use of an electrochemical method to interconvert between the metal oxidation states.

**Scheme 24:**
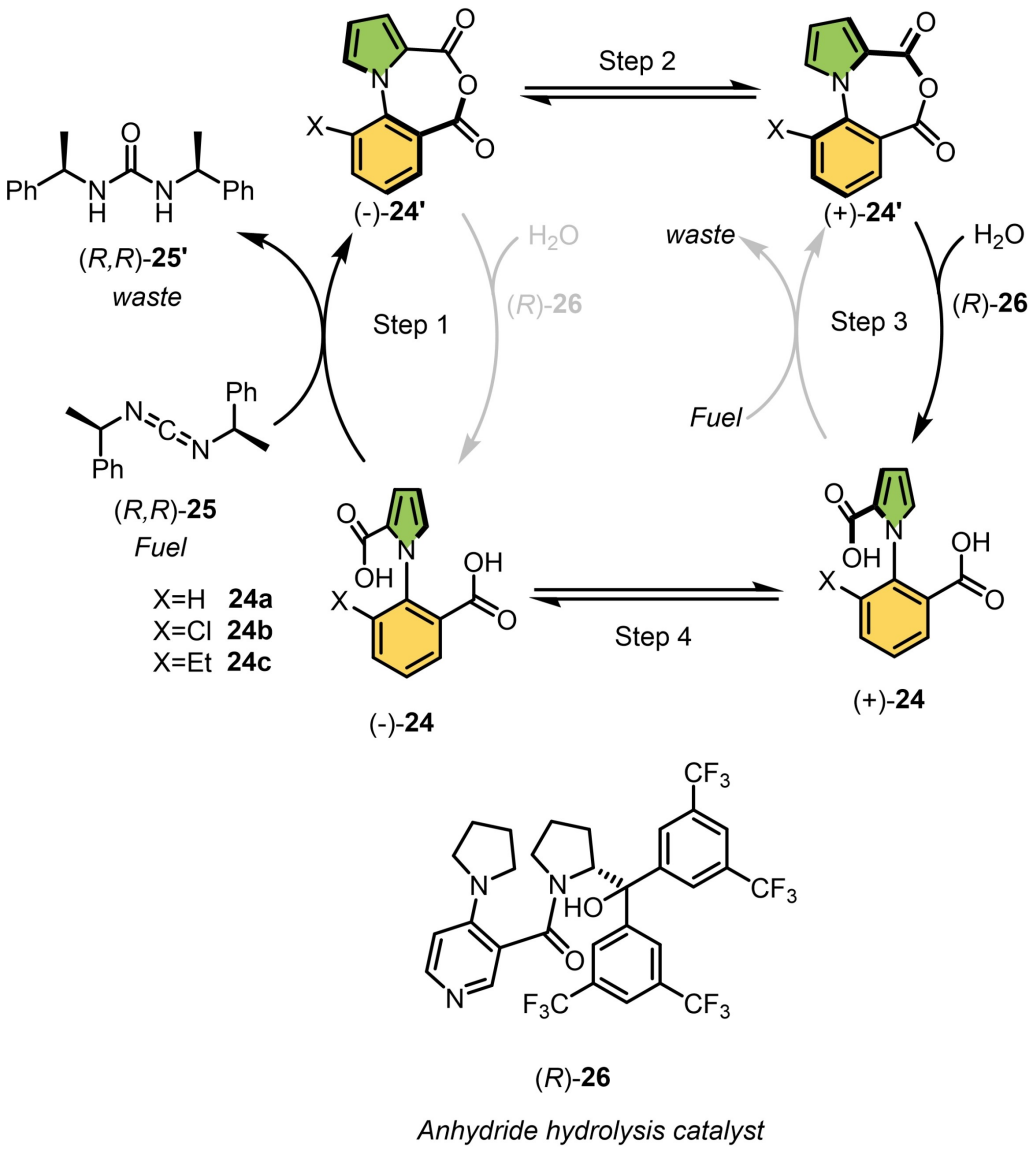
The first autonomous engine to cause 360° rotation around a single bond by catalysis.1‐Phenylpyrrole‐2,2′‐dicarboxylic acid **24** can continuously convert energy from a chemical fuel to cause repeated 360° rotation of the two aromatic rings through the continuous intramolecular anhydride formation between the rings and hydrolysis of the anhydride. The chiral fuel **25** and the hydrolysis catalyst **26** are used to create a directional bias leading to a net rotation around the N−C bond.^[101]^

As discussed earlier, molecular motors powered by chemical conversion could serve a variety of functions as molecular engines in nanotechnology, provided they operate autonomously, i.e., the rotor unit rotates 360° continuously as long as fuel is supplied. According to a recent study by Leigh and co‐workers, an arylpyrrole‐2,2′‐dicarboxylic acid derivative **24 a** can autonomously rotate about its C−N bond when fueled with carbodiimide (Scheme 24).^[101]^ The key steps in their design are the formation of a cyclic bridged anhydride **24′** (step 1, Scheme 24) and its simultaneous hydrolysis using an organocatalyst (step 3, Scheme 24). Choosing the appropriate handedness of the chiral fuel and the hydrolysis catalyst creates a directional bias (e.g., (−)‐**24** to (−)‐**24′** and (+)‐**24** to (+)‐**24′**; gray arrows Scheme 24)) so that rotation preferentially occurs in one direction. According to their proposed mechanism, the autonomous process begins with the conversion of the diacid **24** into the corresponding anhydride, **24′**, using carbodiimide as fuel, producing urea as a waste (step 1). In the presence of chiral fuel (*R,R*)‐**25**, one atropisomer might react faster than the other ((−)‐**24** reacts faster than (+)‐**24** in a diastereoselective cyclization; Scheme 24), which gives some directionality. In the second step, the newly formed bridged anhydride **24′** would be configurationally labile in nature and should undergo a rapid atropisomerization ((−)‐**24′**⇌(+)‐**24′**; Scheme 24). The anhydride **24′** is then hydrolyzed using a chiral organocatalyst (*R*)‐**26**, giving rise to the diacid (+)‐**24** through a dynamic kinetic resolution process, “locking” the conformation of the motor molecule. In the final step, thermal rotational isomerization of (+)‐**24** can take place, completing the 360° rotation of the C−N bond and a motor cycle. Experimentally, the authors were not able to demonstrate that diacid **24 a** is an autonomous directed motor, as claimed, because of the lack of experimental techniques available and free rotation around its C−N bond. However, they have elucidated the fundamental mechanism of each step during the rotational cycle with the analogous compounds **24 b** and **24 c**, as they exhibit high configurational stability at room temperature. For further investigation, they used (±)‐**24 c** as a model substrate for the continuous intramolecular anhydride formation and hydrolysis of the anhydride. In fact, anhydride formation using the chiral fuel (*R,R*)‐**25** and its subsequent hydrolysis in the presence of the chiral organocatalyst (*R*)‐**26** led to the enantioenriched compound **24 c** with an e.r. of 71 : 29, meaning that there is a bias in the rotation direction. Finally, to test whether the carboxylic acid present in pyrrole rotator only passes through the substituent at the 6‐position of the aryl ring, the rotational barriers for **24 a**–**c** were calculated. Gas‐phase DFT studies showed that the barrier for atropisomerization of carboxylic acids passing the X‐ substituents of the stator is much lower due to the steric hindrance between the two carboxylic acid groups. Moreover, the experimental fact that **24 c** racemizes much more slowly than **24 b** confirmed that racemization is caused by rotation of CO_2_H passing the ‐X substituent. This chemical cycle consumes more than 97 % of the fuel molecules, with directional bias up to 71 : 29 when chirality‐matched fuels and additives are used. During this autonomous catalytic cycle, the motor **24** acts as a catalyst for the hydrolysis of carbodiimide, which continues as long as the fuel molecules are present. The motor rotates approximately every three hours and makes a mistake in direction every three or four turns. Despite the fact that this system is still far from a perfect molecular ratchet motor, it addresses the important question of how to achieve autonomous and continuous rotary motion. The different approaches to chemically fueled unidirectional rotary motion have revealed the basic principles for further optimizations and novel designs that may result in genuine catalytic motors and stimulate future applications of the chemically driven molecular motors.

## 6 Conclusion and Prospective

During three decades of combined efforts by researchers, several ingenious examples of chemically driven molecular switches and rotors based on atropisomers have been reported. They have all exploited chemical interactions such as hydrogen bonding, pH response, metal coordination, conformational change (rotation) due to bond formation and cleavage in dynamic systems. The introduction of responsive features allows molecular motion to be controlled by external chemical stimuli. Next, these fundamental studies laid the groundwork for the future development of molecular machines, and four examples of chemically driven functional rotatory molecular motors which demonstrate repetitive unidirectional 360° rotation have been successfully constructed to date. Although significant progress has been made in this field, and these proof‐of‐principle systems will spark novel designs, there are still major fundamental challenges to be addressed. Like biological motor proteins which are powered by ATP hydrolysis or a proton gradient, the ultimate goal of artificial chemically driven molecular machines is to develop a continuously and autonomously rotating motor as we discussed in the introduction section. Based on the strategies discussed in this Review, we provide two possible parallel routes for further investigation and future developments towards autonomous molecular motors.

1. *Unidirectional molecular rotors*: One strategy is to modify the design of molecular rotors. Rotors undergo constant but random rotation due to the lack of directional bias in the movement of the components. Following the example of molecular motors, introduction of a chiral group in the close vicinity of the rotation axle of a rotor coupled to a chemical transformation to power the rotation is expected to provide full unidirectionality to molecular rotors.
2. *Continuous molecular rotatory motors*: The other way is to make the motion of molecular motors continuous. Most of the current examples of molecular motors show unidirectionality in rotation, but multiple steps are necessary to achieve full rotation. By reducing the number of reaction steps as in Leigh's work or by using a catalytic (transition‐metal‐catalyzed motor) or redox approach (electrochemically driven motor) as shown in Figure 3
  **Figure 3:**
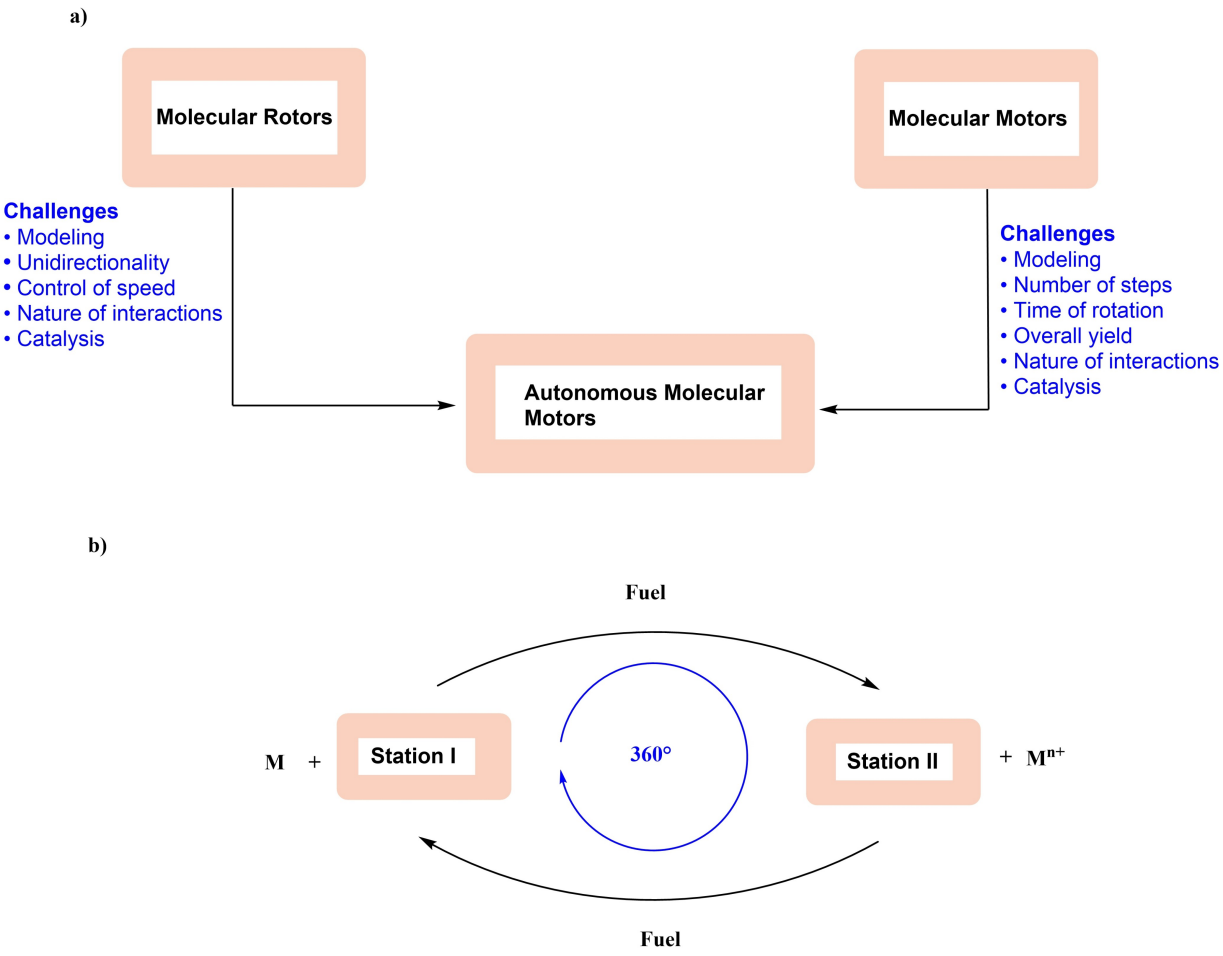
Challenges and prospectives. a) A roadmap outlining some of the challenges, in transforming molecular motors and rotors into autonomous molecular motors. b) A proposed blueprint of a catalytic molecular motor.
  b, autonomous motion might be accomplished.

Specific key challenges are also listed in Figure 3.

*Directionality*: The major part of the work presented in this Review is dedicated to the acceleration of the rotor speed through a chemical modification. However, the rotational direction is not controlled. The design of alternative rotor structures where the nondirectional rotation of an existing rotor could be transformed into a unidirectional autonomous rotation, possibly using a chiral auxiliary or chiral catalyst (cf. ref. [101]), building on the advances in asymmetric catalysis, deserves to be broadly explored.

*The number of steps for one full rotation*: The major drawback of current designs of chemically driven molecular motors is the number of steps required to achieve one single rotation. Light‐driven molecular motors based on overcrowded alkenes are so far rather unique as they undergo, upon irradiation, continuous rotation. On the other hand, an autonomous chemically driven catenane‐based rotary system^[88f]^ and one example based on a biaryl system^[101]^ have been recently reported. Utilizing the concepts discussed in this Review, there must be ample opportunities to design next generations and more sophisticated biaryl motors.

*Time of rotation*: In addition to the number of chemical steps, the time required for a complete rotation is also a major challenge because isolation of intermediates is time‐consuming. For a faster process, is it possible to design a responsive molecular system in which manipulation of the noncovalent interactions by a chemical stimulus results in directed rotation? One can take advantage of all the rotors discussed in this Review, which can undergo rotation through chemical interactions such as hydrogen bonding, pH response, metal coordination, and dynamic bond formation and cleavage. None of these molecular rotors require isolation of the intermediates. If chemical transformation can be coupled with control of noncovalent interactions, continuous rotation may be possible.

*Catalytic motors*: In biological molecular machines, the majority of the autonomous processes such as assembly, transport, and motion are mainly governed by chemical catalysis. Despite the tremendous advancement in the field of metal catalysis, only very limited approaches towards fully functional catalytic rotatory motors have been reported so far. Can we ultimately build a catalytic motor, where one can switch in a directional manner from one station to the other autonomously using a single metal catalyst and fuel as shown in Figure 3b?

*Chemically driven molecular motors to control function*: Molecular switches and motors are an intriguing class of molecules that control molecular functions by altering the properties of a system in response to an external stimulus. The ultimate challenge is to attain those responsive properties and adaptive function using their unique directional rotatory motion. As discussed earlier, it is fascinating to observe how nature controls movement by the conversion of chemical energy into mechanical energy and amplifies motion along length scales. In this context, notable progress has been made using light‐driven molecular motors.^[102]^

*Application of chemically driven motors on surfaces*: Although chemically driven molecular motors are designed to operate in solution, they have had to be mounted on surfaces for their use as nanomachines.^[103]^ In solution, the directed motion of a molecular motor is dissipated by the nondirectional thermal motion of the molecules. However, when molecular motors are grafted on a surface, translational motion in three dimensions is inhibited and an absolute net rotation can be achieved. Meanwhile also synchronization might be possible. The key challenges in developing such systems is first to attach molecular motors on the surface using an appropriate anchoring group without interfering the rotational cycle.

*Biocompatibility*: Beyond all the challenges mentioned above, one major point that remains is to develop systems which can perform tasks in biocompatible aqueous media in order to allow potential biomedical application.

Finally, as we discussed in this Review, we now have better understanding of how to design rotors and motors to control molecular motion about a single bond. However, for a wider range of applications of these machines further improvements are essential. The development of a molecular motor where all the rotational steps can be done fast, in particular with the aid of minimal chemical steps, in a repetitive and continuous manner, without compromising directionality, tolerating various functions and as part of more complex multifunctional (mechanical) systems, is of key importance for future developments. Furthermore, the proof of principle used in the development of switches, rotors, and motors can provide a major source of inspiration for the development of novel designs of highly desired fully autonomously operating chemically driven rotatory molecular motors.

## Conflict of interest

The authors declare no conflict of interest.

## Biographical Information

*Anirban Mondal grew up in Kolkata, in the eastern part of India. He did his master's thesis at the Indian Institute of Technology Bombay, Mumbai, under the supervision of Prof. Debabrata Maiti and Rodney A. Fernandes. In 2016, he joined the group of Prof. Ben L. Feringa at the University of Groningen for his doctoral research. He recently completed his PhD focusing on various chirality transfer processes in the development of chemically driven molecular machines and asymmetric catalysis*.

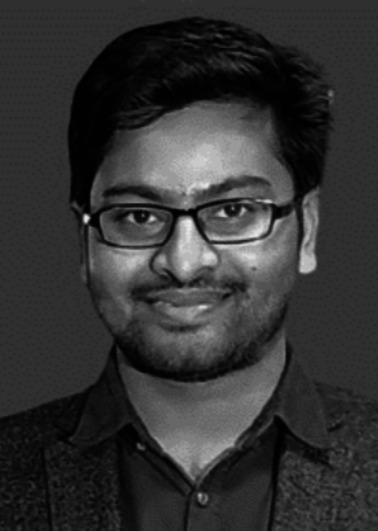

## Biographical Information

*Ryojun Toyoda obtained his PhD from the University of Tokyo (Japan) under the guidance of Prof. Hiroshi Nishihara in 2020. He then joined the group of Prof. Ben L. Feringa at University of Groningen as a postdoctoral researcher with support from a JSPS overseas fellowship. He worked on molecular machines and smart materials there. In 2022, he was appointed as an assistant professor at Tohoku University (Japan) where he is working on coordination chemistry, nanoscience, photofunctional molecules, molecular machines, and single‐molecule nanomaterials*.

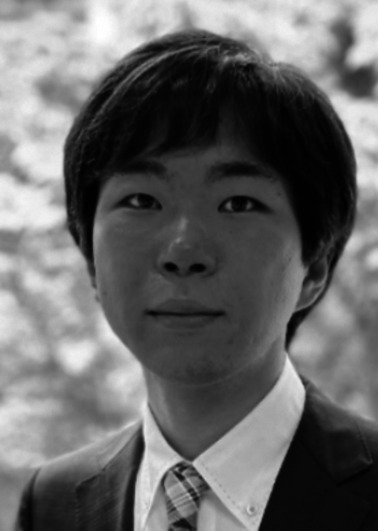

## Biographical Information

*Romain Costil studied Organic, Bio‐organic and Therapeutic Chemistry at the Ecole Nationale Supérieure de Chimie de Mulhouse (France), where he was awarded a Diplome d'ingénieur in 2014. He then joined the group of Prof. Jonathan Clayden at University of Bristol (UK) for a MSc project in foldamer chemistry, followed by PhD research on the synthesis and properties of a novel class of atropisomers. Following postdoctoral research in the Feringa lab working on novel molecular motors, he stayed as research manager in the same group*.

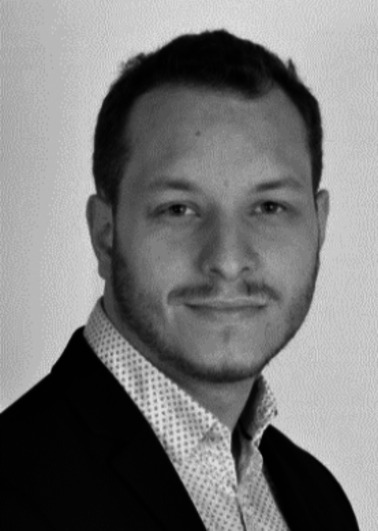

## Biographical Information

*Ben L. Feringa obtained his PhD degree in 1978 at the University of Groningen in the Netherlands under the guidance of Prof. Hans Wynberg. After working as a research scientist at Shell he was appointed full professor at the University of Groningen in 1988 and named the distinguished Jacobus H. van't Hoff Professor of Molecular Sciences in 2004. He was elected foreign honorary member of the American Academy of Arts and Sciences and member of the Royal Netherlands Academy of Sciences. His research interests include stereochemistry, organic synthesis, asymmetric catalysis, molecular switches and motors, photopharmacology, self‐assembly and nanosystems*.